# SMARCB1 deletion in atypical teratoid rhabdoid tumors results in human endogenous retrovirus K (HML-2) expression

**DOI:** 10.1038/s41598-021-92223-x

**Published:** 2021-06-18

**Authors:** Tara T. Doucet-O’Hare, Brianna L. DiSanza, Catherine DeMarino, Abigail L. Atkinson, Jared S. Rosenblum, Lisa J. Henderson, Kory R. Johnson, Jeffrey Kowalak, Marta Garcia-Montojo, Sariah J. Allen, Brent A. Orr, Mariarita Santi, Tongguang Wang, Saeed Fathi, Myoung Hwa Lee, Kevon Sampson, Wenxue Li, Zhengping Zhuang, Avindra Nath

**Affiliations:** 1 Section of Infection of the Nervous System, Disorders and Stroke (NINDS), National Institute of Neurological, National Institutes of Health (NIH), Bldg 10; Room 7C-103; 10 Center Drive, Bethesda, MD 20892 USA; 2 Neuro-Oncology Branch, National Cancer Institute (NCI), Bethesda, USA; 3 Bioinformatics Section, National Institute of Neurological Disorders and Stroke, Bethesda, USA; 4 Clinical Proteomics Unit, National Cancer Institute (NCI), Bethesda, USA; 5 Department of Pathology, St. Jude’s Children’s Research Hospital, Memphis, USA; 6 Department of Pathology, Perelman School of Medicine, Children’s Hospital of Philadelphia, University of Pennsylvania, Philadelphia, PA USA; 7 Neural Differentiation Unit, Translational Neuroscience Center, NINDS, Bethesda, NIHMD USA

**Keywords:** Cancer, Paediatric cancer, Genetics, Epigenomics

## Abstract

Atypical Teratoid Rhabdoid Tumor (AT/RT) is a rare pediatric central nervous system cancer often characterized by deletion or mutation of *SMARCB1*, a tumor suppressor gene. In this study, we found that SMARCB1 regulates Human Endogenous Retrovirus K (HERV-K, subtype HML-2) expression. HML-2 is a repetitive element scattered throughout the human genome, encoding several intact viral proteins that have been associated with stem cell maintenance and tumorigenesis. We found HML-2 env expression in both the intracellular and extracellular compartments in all AT/RT cell lines (n = 4) and in 95% of AT/RT patient tissues (n = 37) evaluated. *SMARCB1* knock-down in neural stem cells (NSCs) led to an upregulation of HML-2 transcription. We found that SMARCB1 binds adjacent to the HML-2 promoter, repressing its transcription via chromatin immunoprecipitation; restoration of *SMARCB1* expression in AT/RT cell lines significantly downregulated HML-2 expression. Further, targeted downregulation of HML-2 transcription via CRISPR-dCas9 coupled with suppressor proteins led to cellular dispersion, decreased proliferation*,* and cell death in vitro. HML-2 knock-down with shRNA, siRNA, and CRISPR-dCas9 significantly decreased Ras expression as measured by qRT-PCR, suggesting that HML-2 modulates *MAPK/ERK* signaling in AT/RT cells. Overexpression of *NRAS* was sufficient to restore cellular proliferation, and MYC, a transcription factor downstream of *NRAS*, was bound to the HERV-K LTR significantly more in the absence of *SMARCB1* expression in AT/RT cells. We show a mechanism by which these undifferentiated tumors remain pluripotent, and we demonstrate that their formation is aided by aberrant HML-2 activation, which is dependent on *SMARCB1* and its interaction with MYC.

## Introduction

Atypical teratoid rhabdoid tumor (AT/RT) is a rare embryonal central nervous system (CNS) cancer diagnosed most often on the basis of biallelic loss of *SMARCB1* (SWI/SNF Related, Matrix Associated, Actin Dependent Regulator Of Chromatin, subfamily B, Member 1), a master chromatin regulator which is essential during development^1^. Mortality remains high in patients with AT/RT due to limited treatment options such as tumor resection, chemotherapy, and in a select population, radiation therapy^2^. AT/RTs are comprised of undifferentiated cancer cells characterized by expression of epithelial, mesenchymal, and neuroectodermal markers and loss of *SMARCB1* expression^1^. This cancer is most common in children under 3 years of age and is largely due to a failure of proper development, neuronal cell migration, and cell differentiation^3,4^.

*SMARCB1* is one of the core proteins in the SWItch/Sucrose Non Fermentable (SWI/SNF) chromatin remodeling complex, which contributes to conformational changes in the nucleosome, altering DNA-histone binding, and resulting in transcription factor access to gene promoters^4^. When *SMARCB1* is absent, there is residual function of *SMARCA2/4*, the ATPase subunit of the chromatin remodeling complex; however regulation of enhancers which are key for development and differentiation are lost^4^. The activity of *SMARCA2/4* is present at a barely detectable level and occupies super enhancers, areas with high transcription factor density, resulting in the maintenance of a stem-cell identity^4^. *SMARCB1* loss of expression has different effects depending on the timing of the inactivation. In a study using conditional knock out mice, the early loss of *SMARCB1* expression in neural crest cells caused the development of rhabdoid tumors^5^. A loss of both *NF2* (neurofibromatosis 2) and *SMARCB1* at a later stage of development in the Schwann cell lineage, led to the development of schwannomas^5^. In addition, mutations in *SMARCB1* have been connected to brain midline defects such as glial aberrations leading to agenesis of the corpus callosum in the conditional *SMARCB1* knockout mouse^6^. The defects observed in the *SMARCB1* conditional knockout mouse are largely due to ineffective neuronal migration and neuronal maturation^6^.

*SMARCB1* is a transcriptional repressor of integrated Human Immunodeficiency Virus (HIV) long terminal repeat (LTR) promoter^7^. When *SMARCB1* expression is depleted, HIV transcription increases; further, the reappearance of *SMARCB1* expression results in decreased viral transcription and epigenetic silencing^7^. Long terminal repeats are a consequence of retroviral integration and also flank Human Endogenous Retroviruses (HERVs) which comprise about 8% of the human genome and were acquired throughout human evolution^8,9^. Each integrated retrovirus has an LTR, which acts as the promoter for the HERV; LTR activity is regulated by methylation of CpGs in the LTR^10–12^ and by many transcription factors that can bind to its sequence such as C-MYC^13^. Currently it is not known how *SMARCB1*-mediated chromatin remodeling around the LTR regulates endogenous retroviral activity. In the human genome, transcription factor binding, CpG methylation, and chromatin remodeling all work in conjunction to alter expression of endogenous retroviruses^14^. There are only a few chromatin remodeling proteins which have been linked to endogenous retroviral suppression in humans such as *SETDB1* and *ATRX*^15,16^.

When chromatin is tightly closed, it is difficult for transcription factors to access DNA; further, targeted binding of transcription factors can prompt chromatin remodeling resulting in an accessible promoter^17^. The SWI/SNF complex, including SMARCB1, does not bind to DNA based on sequence^18^; rather, the N-terminus of SMARCB1 interacts with acidic transcription sites and the SWI/SNF complex binding is facilitated by ultrastructural interaction and nucleosome positioning^17,19^. SMARCB1 binds C-MYC protein under non-pathogenic conditions; when SMARCB1 is absent, there is more C-MYC freely available to bind transcription factor sites^13^. C-MYC has not been definitively shown to bind an HERV-K LTR; however, many HERV-K LTRs in the human genome harbor multiple C-MYC binding sites^20^.

HERVs originated from exogenous viruses that were incorporated into the genome of the host germline and thereby maintained in subsequent generations^21^. The most recent additions to the genome are LTR5_Hs (Hs for human specific) and HERV-K (subtype HML-2)^22,23^; these gene features are relevant to disease phenotype because they often encode one or more intact viral proteins^24^. Normally, HML-2 expression is tightly controlled both temporally and spatially in stem cells and during development^25^. Further, HML-2 RNA and proteins are not highly expressed in differentiated cells or tissue; if expressed, they induce cytotoxicity in non-dividing cells such as neurons^26^.

Expression of HML-2 envelope (env) confers stem cell like features in dividing cells if not silenced^27^. Improper regulation of HML-2 protein expression in dividing cells may result in uncontrolled growth that leads to cancer^28^. Further, it has been shown that *ras* is downregulated in concordance with HML-2 downregulation in vivo*,* suggesting that its expression may be regulated by HML-2^29,30^.

Herein, we sought to understand the role of *SMARCB1* in mediating endogenous retrovirus expression in the undifferentiated embryonal cancer AT/RT. Further, we evaluated the membrane for vesicle production and neurite outgrowth, which we expected to be abnormal given the undifferentiated and neural crest-like cells that comprise these tumors^31–33^. We hypothesize that loss of *SMARCB1* chromatin remodeling coupled with enhanced C-MYC binding to the HML-2 LTR results in constitutive expression of HML-2 elements, allowing cells to maintain a stem-cell like identity and persist as this developmental tumor.

## Results

### Detection of HERV-K (HML-2) in AT/RT tumors

**Figure 1:**
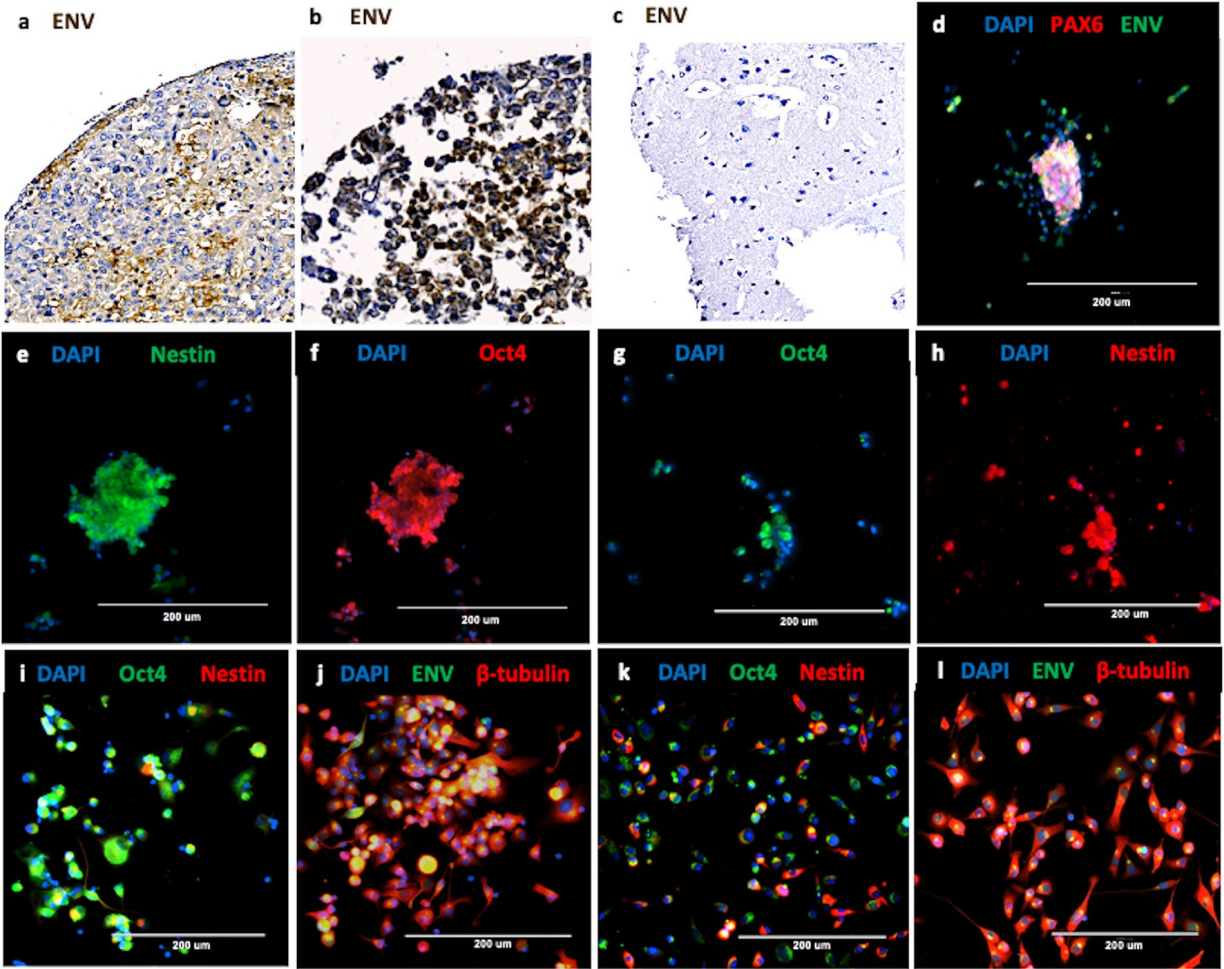
Immunostaining and Immunohistochemistry of patient-derived AT/RT cells and tissues. (**a**,**b**) Two resected patient AT/RT demonstrate strong immunostaining with HML-2 Envelope (Env) monoclonal antibody (brown). (**c**) Cerebrum from a normal brain is negative when stained with the same antibody. (**d**–**l**) AT/RT cell lines express markers of multiple stages of differentiation; representative images are shown. (**d**) CHLA 02, a patient derived AT/RT cell line, expresses Pax6 (red), a marker for neuroectoderm, merged with immunostaining for HML-2 Env (green). (**e**)*:* This cell line also expresses Nestin (green), a marker of neural stem cells. (**f**) The same cell line expresses Oct4 (red), a pluripotency marker. (**g**,**h**) CHLA 04 cells, another AT/RT line, express Oct4 (green) and Nestin (red). (**i**) CHLA 05 cells also express Oct4 (green) and Nestin (red). (**j**) The same cell line also expresses βIII tubulin (red), a marker for neurons and HML-2 envelope (green). (**k**) CHLA 06 cells also express Nestin (red) and Oct4 (green). (**l**) The same cell line also expresses βIII tubulin (red) and HML-2 Env (green). Nuclei are stained with DAPI (blue). For IHC images, slides were scanned with a OptraScan Digital Pathology Scanner and processed in Adobe Photoshop for incorporation into the figure. For immunofluorescent images, an EVOS fluorescence microscope (AMG) was used. Images were acquired with the native software installed on the EVOS microscope and processed in Microsoft PowerPoint (https://www.thermofisher.com/us/en/home/technical-resources/software-downloads/evos-fl-cell-imaging-system.html).

AT/RT tissues derived from 35 of 37 (95%) patients demonstrated clusters of cells strongly expressing HML-2 env protein as measured by a monoclonal env antibody immunohistochemistry on a tissue microarray (Fig. 1, Supplemental Table S1, Supplemental Fig. S1). No env expression was observed in the normal cerebellum or in non-AT/RT brain samples (Fig. 1C, Supplemental Table S1, Supplemental Fig. S1). All ten of the AT/RT tissues originating from recurring tumors expressed HML-2 env (Supplemental Table S1). Placenta was stained as a positive control^34^ due to its mixed composition of mesenchymal and progenitor cells (Supplemental Fig. S1).

### Characterization of AT/RT cell lines

Four AT/RT cell lines CHLA 02, CHLA 04, CHLA 05, and CHLA 06, were used in this study. All four cell lines lacked *SMARCB1* expression^35,36^ and expressed markers indicative of pluripotent cells, including: HML-2 env and *OCT4* (a marker for stem cells), *Pax6* (a neuroectodermal marker), *NES* (a neural stem cell marker, nestin), and *ΒIII-tubulin* (a neuronal marker) (Fig. 1d–l). Immunocytochemical characterization revealed that the AT/RT cells are a mixed population at various stages of differentiation but they all express HML-2 env in the plasma membrane (Fig. 1). In previous publications, identification of subgroups of AT/RT tumors and tumor cell lines were described with regard to genetic and epigenetic expression and specific therapeutic treatment responses^36,37^. CHLA 02, CHLA 04, and CHLA 05 cell lines were all classified as group 1 neurogenic tumors while CHLA 06 cell line was classified as group 2 due to differences in methylation patterns and distinct clinical and genotypic features^36,37^. With an updated analysis of AT/RT subgroups, group 1 neurogenic tumors have been classified as ATRT-SHH, sonic hedgehog, and group 2 tumors fall into either an ATRT-MYC, MYC proto-oncogene, or a ATRT-TYR, tyrosine, subtype^37^. Even though one of the four cell lines was classified as belonging to a different group, all four cell lines expressed HML-2 env suggesting env expression may be a broad marker for AT/RT tumors.

### Cellular and extracellular vesicles express of HML-2 env

**Figure 2:**
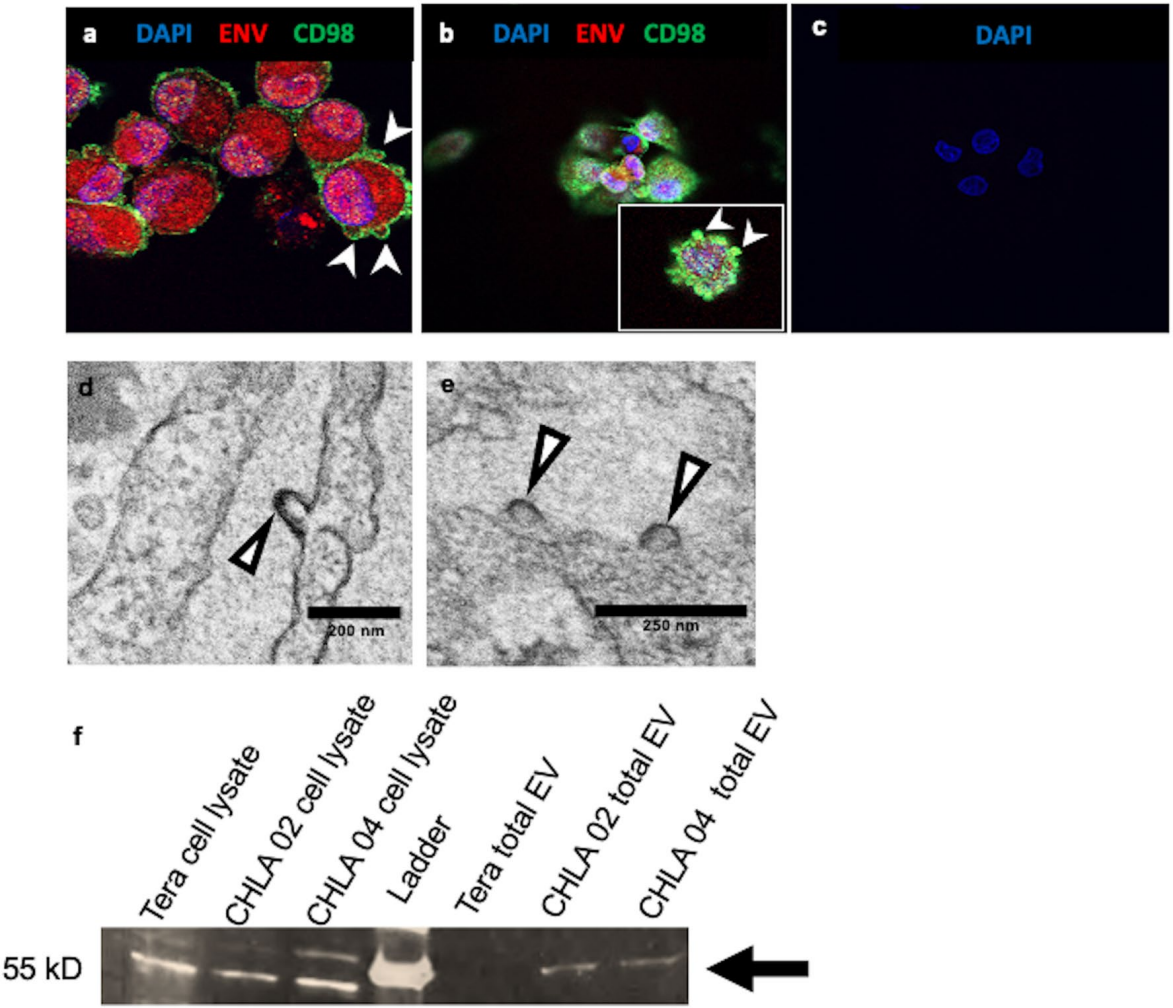
Extracellular Vesicles are released from CHLA 02 and CHLA 04 cells and contain HML-2 Envelope (Env). (**a**,**b**) AT/RT cells release extracellular vesicles contain HML-2 Env*.* (**a**) Immunostaining CHLA 02 cells with HML-2 env polyclonal antibody (surface unit) (red) and *CD98* (green), a cell-surface marker, shows Env is expressed in extracellular vesicles enclosed by the plasma membrane. Cell nuclei are stained with DAPI (blue); (**b**) CHLA 04 cells similarly immunostained; magnification is at 63X. (**c**) CHLA 02 cells incubated with secondary antibody only as a control for nonspecific immunostaining. (**d**,**e**) Electron microscopy shows extracellular vesicles forming on plasma membrane of CHLA 02 AT/RT cells (arrow heads). (**f**) Cell lysates from Tera, CHLA 02, and CHLA 04 cells express HML-2 env (left three lanes) while only purified extracellular vesicles from CHLA 02 and CHLA 04 express HML-2 env (three right lanes) on immunoblot (arrow). A fluorescent Zeiss confocal microscope (LSM510) was used to acquire images processed with Zeiss LSM 5 Image Browser and Microsoft PowerPoint for figure creation (https://www.embl.de/eamnet/html/downloads.html). The gel image has been cropped to focus on the bands of interest, the full gel image can be found in the supplementary material. Gel Image was taken with a FluorChem E system (https://www.proteinsimple.com/fluorchem.html) and images were processed with ImageJ and Microsoft PowerPoint for relative densitometry quantification and display. Electron microscopy images were taken with a JEOL 1200 EXII Transmission Electron Microscope (AMT digital camera system).

HML-2 env was identified in the cytoplasm and the plasma membrane by confocal microscopy in the CHLA 02 and CHLA 04 lines (Fig. 2a–c). Env was found to colocalize with CD98, a putative receptor for HML-2 env^35^. We further evaluated the CHLA 02 cells by electron microscopy and found no evidence of typical viral budding in approximately 100 cells. However, membrane-like structures resembling exocytosis of vesicles were noted (Fig. 2d,e). These appear to correspond to vesicles of similar morphology seen on confocal imaging to contain HML-2 env protein (Fig. 2). We confirmed that the atypical membrane structures observed by EM and confocal imaging contain HML-2 env protein by immunoblotting of enriched extracellular vesicles from cell free media via Nanotrap particles (Fig. 2f).

### Regulation of HML-2 repetitive elements by SMARCB1

**Figure 3:**
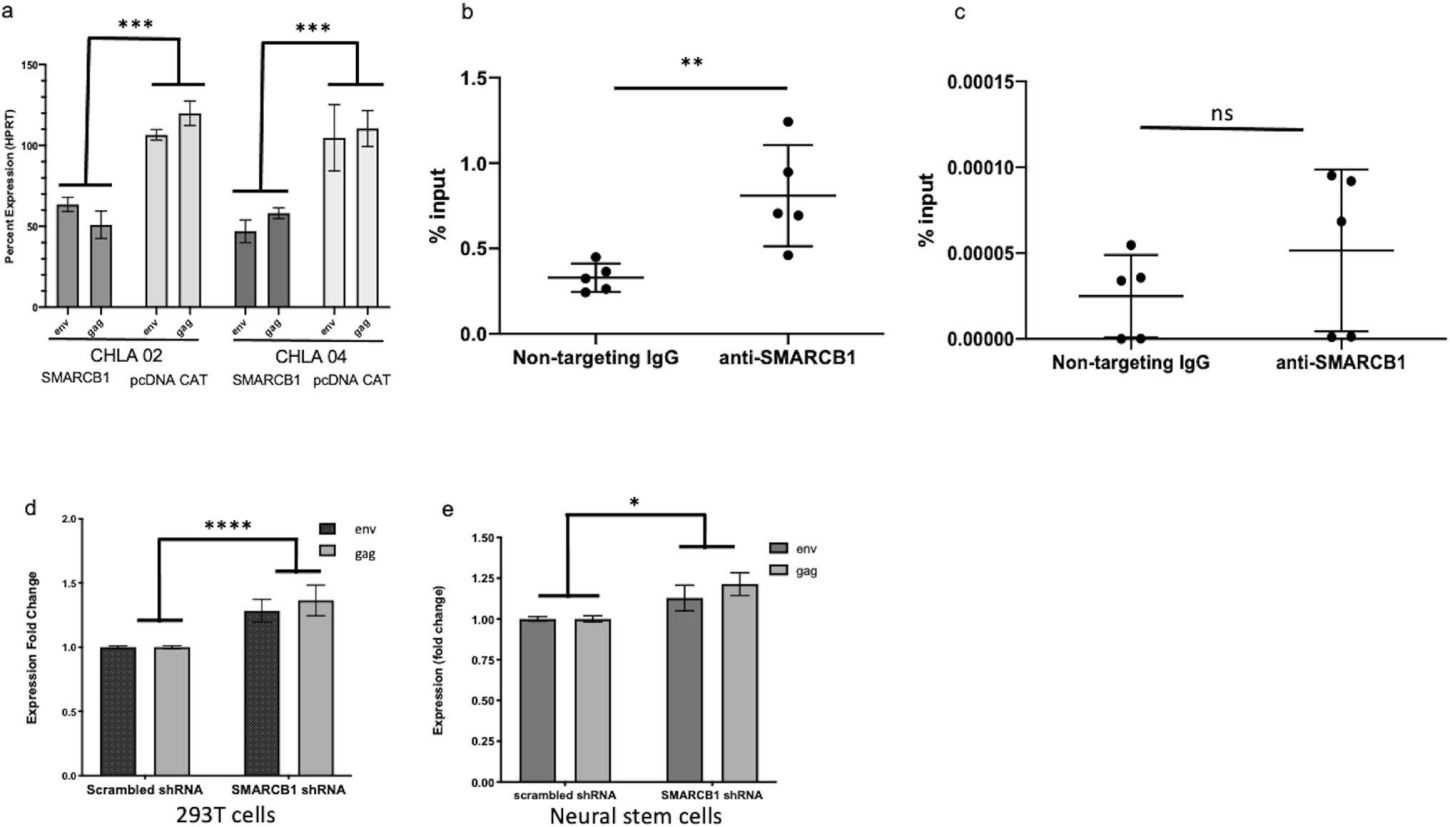
SMARCB1 regulates HERV-K (HML-2) env expression. (**a**) Restored *SMARCB1* expression in CHLA 02 and CHLA 04 AT/RT cell lines results in downregulation of HML-2 transcription measured at 48 h by qRT-PCR. (**b**,**c**) SMARCB1 binds the HML-2 LTR significantly more than the promoter of control gene, *HPRT*. (**b**) *SMARCB1* transfected 293 T cells show a significantly greater proportion of HML-2 LTR bound to *SMARCB1* following immunoprecipitation compared to control, non-targeting IgG. (**c**) *SMARCB1* transfected 293 T cells show no significant difference between non-targeting IgG bound to genomic *HPRT* and SMARCB1 bound to genomic *HPRT*. Percent input is a normalized value with input set to 100% (e.g. % input = 100*2^(input Ct − immunoprecipitated chromatin Ct). Ct is cycle threshold. (**d**,**e**) *SMARCB1* knockdown results in increased transcription of HML-2 transcripts as measured with qRT-PCR. (**d**) HML-2 transcripts in 293 T cells transfected with scrambled shRNA control compared to shRNA targeting *SMARCB1* at 24 h as measured by qRT-PCR. (**e**) HML-2 transcripts in neural stem cells transfected with scrambled shRNA control are significantly higher compared to transcripts in cells transfected with shRNA targeting *SMARCB1* at 48 h. (qRT-PCR) Data was entered into Prism v9 for graph creation. [Error bars represent SEM. Statistics in Supplemental Table S6].

Overexpression of *SMARCB1* in CHLA 02 and CHLA 04 cells led to significantly reduced transcription of HML-2 genes, suggesting *SMARCB1* regulates HML-2 transcription (Fig. 3a). We observed increased binding of SMARCB1 to the HML-2 LTR compared to the housekeeping gene *Hypoxanthine–guanine phosphoribosyltransferase* (*HPRT)* (Fig. 3b,c). HML-2 transcription increased following knockdown of *SMARCB1* in both 293 T cells (Fig. 3d) and in NSCs (Fig. 3e).

### RNA Sequencing Analysis of AT/RT Cell Lines

RNA expression profiles determined by next generation sequencing supported the immunohistochemical findings (Fig. 1), indicating that each tumor originated from cells at different stages of development/differentiation. We found HML-2 repetitive elements within highly expressed genes in these cell lines. Two groups of highly expressed genes containing either sequences of HML-2 internal coding sequences (HML-2 int) or LTR5_Hs were found by DAVID functional clustering gene ontology analysis^38^. The first group contained genes related to Krüppel associated box (KRAB) and zinc finger proteins both of which play roles in transcriptional regulation (Supplemental Table S2). The genes in the second group were affiliated with the *neuroblastoma breakpoint family* (*NBPF*) (Supplemental Table S2); genes in this family, such as putative tumor suppressor *NBPF1*, which is highly expressed in the brain, have repetitive structures containing both intragenic and intergenic sequences^39^.

Proteomic analysis of these cell lines confirmed the high expression observed by RNA sequencing analysis and the immunohistochemistry (IHC). Two groups of highly expressed proteins were found after analysis with DAVID of the CHLA 02 and CHLA 04 AT/RT cells by liquid chromatography mass spectrometry (LCMS). Both cell lines expressed proteins associated with the cellular immune response to viral proteins, in addition to neural stem cell marker (nestin), neuronal marker (βIII-tubulin), and glial cells (vimentin), which clustered as the first group (Supplemental Table S3). One of the proteins highly expressed in CHLA 02 and CHLA 04 AT/RT cells is adenovirus early region 1B associated protein 5, a protein known to be activated during adenoviral infection, which has been suggested as a marker for undifferentiated embryonic stem cells^40^. An enrichment of proteins related to cellular adhesion, such as genes affiliated with cadherin cell–cell binding, cell–cell junction, and cell–cell adhesion was detected in the second group (Supplemental Table S3).

### Identification of activated HML-2 loci due to SMARCB1 loss

**Figure 4:**
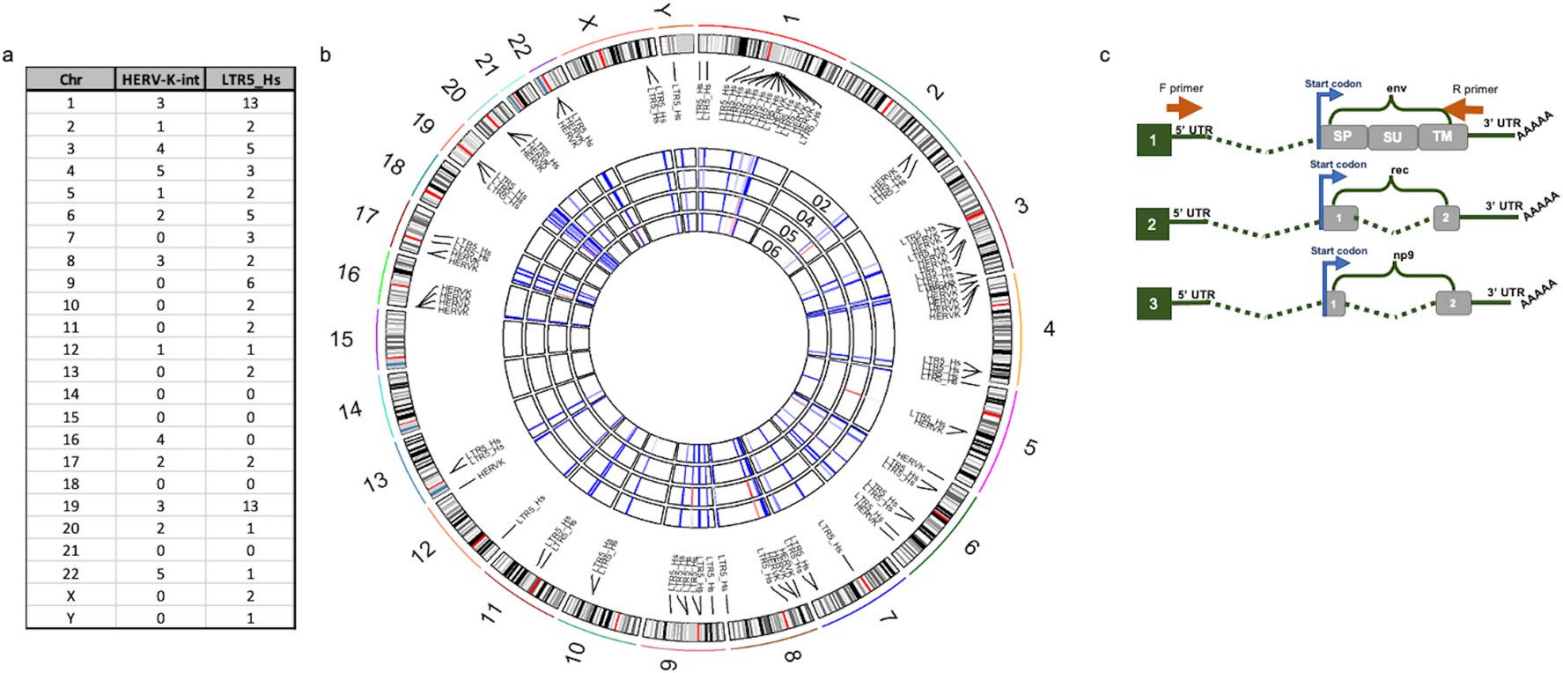
RNA sequencing of AT/RT cell lines. (**a**,**b**) HERV-K (HML-2) internal coding genes and LTR5_Hs are expressed from most chromosomes. (**a**) Table shows quantity of loci expressed on each chromosome (Chr). (**b**) Graphical depiction of HERV-K (HML-2) internal coding genes in (HERV-K-int) and LTR5_Hs in (long terminal repeat 5 human specific) expression in four AT/RT cell lines. The outer ring is comprised of the chromosomes from the human genome with the list of both “HERV-K-int” (internal coding sequence) and LTR5_Hs loci and black lines connecting them to their approximate location on the chromosome. The four rings in the center correspond to the CHLA 02, 04, 05, and 06 cell lines with the 02 cells being the outermost circle and the 06 cells the innermost circle. The segments of the central circles represent the chromosomes; blue and red lines correspond to the level of expression of either the LTR5_Hs or HERV-K (HML-2) labeled at that location (red denotes higher expression, blue denotes less). (**c**) Scheme for PCR amplification and Sanger sequencing validation of HML-2 transcripts. Shows a representation of primer position relative to potential env, rec, and np9 transcripts which are transcribed in the AT/RT cells. The forward primer used for amplifying *env* (1), *rec* (2), and *np 9* (3) transcripts is positioned in the 5’ UTR while the reverse primer is positioned in the 3’ UTR. Because both primers are in the UTRs, env, rec and np9 transcripts can all be amplified with the same primer set. The image in (**b**) was generated with the RCircos package version 1.2.1 (https://bmcbioinformatics.biomedcentral.com/articles/10.1186/1471-2105-14-244).

Initial RNA sequencing analysis from all four AT/RT cell lines revealed the expression of several loci from both LTR5_Hs and HML-2 int (Fig. 4a–c). However, while at least 36 loci expressed transcripts of HML-2 internal sequences, a minority of loci encoded potential proteins (Supplemental Table S4). HML-2 elements were actively expressed from all chromosomes except for 14, 15, 18, and 21 (Fig. 4a,b). The most actively transcribed HML-2 sequences were from chromosomes 1 and 19 each of which had 13 LTR5_Hs transcripts and 3 HML-2 int (Fig. 4a). The expressed transcripts aligning to elements encoding at least one full-length HML-2 gene are listed in Supplemental Table S4. Of these, only the locus on Chr19q11 encodes full length and potentially functional env protein (highlighted in Supplemental Table S4). Using a second analysis method, the TEtranscripts pipeline^41,42^ (Supplemental Fig. S3), transcripts were detected from all chromosomes except 21 (Supplemental Fig. S3a). Three loci identified by the TEtranscripts pipeline were capable of producing full length env protein: Chr7p22.1a, Chr7p22.1b, and Chr19q11 (Supplemental Fig. S3b, highlighted in Supplemental Table S4). Transcripts originating from both Chr19q11, Chr7p22.1a, and Chr7p22.1b were confirmed with RT-PCR and Sanger sequencing (Supplemental Table S5). The loci on chromosome 7 are a pair of duplications^24^ whose sequences are nearly identical, and both contain an intact *env* gene. Both methods suggested env expression originated from Chr19q11, but with the TEtranscripts pipeline which uses expectation maximization algorithm also suggested the Chr7 loci.

RNA from AT/RT cells was reverse transcribed and then amplified by PCR to confirm the RNA sequencing data (Fig. 4c, Supplemental Table S5). There are two types of HERV-K (HML-2) present in the human genome. Type 1 proviruses possess a 292 bp deletion in the env gene whereas type 2 proviruses have an intact env^14^. Type 1 proviruses express np9, a spliced transcript containing part of the *env* preceding the deletion, and a sequence 3’ of the deletion^14^. Type 2 proviruses can express *env* as well as *rec*, another gene spliced from the beginning of the *env* transcript and an abbreviated 3’ sequence of env. The primers used to amplify *env* were designed to amplify full-length transcript and its smaller spliced products *rec*, or *np9*, (Fig. 4c). Rec and np9 sequences were derived from multiple loci including Chr1p31.1, 1q22, 3q12.3, 3q21.2, 7p22.1a, 7p22.1b, 10q24.1, 11q23.3, 19q11, 19q13.12, and 22q11.21 (Supplemental Table S5). After aligning the Sanger sequences to the human genome and consensus *env* transcript, we compared the data to the expression profile obtained with RNA sequencing (Supplemental Table S4). Only three HML-2 loci which could make a functional full length env protein were identified, namely, Chr19q11, Chr7p22.1a, and Chr7p22.1b.

### Effect of down regulation of HML-2 expression on AT/RT cells

**Figure 5:**
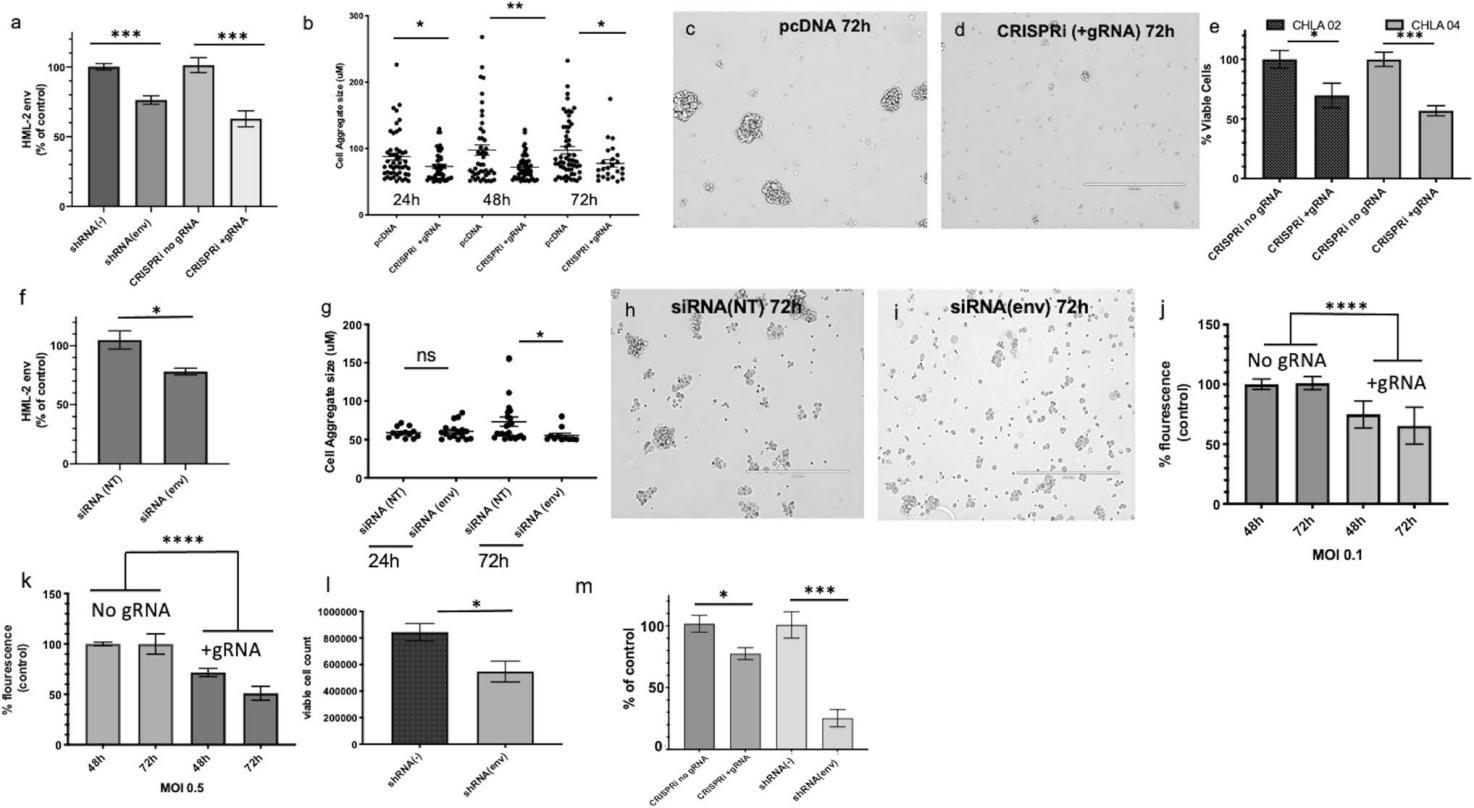
Effect of the Downregulation of HML-2 on AT/RT. (**a**–**d**) A decrease in HML-2 expression leads to reduced cellular aggregates in CHLA 02 AT/RT cells. (**a**) HML-2 env transcript levels in CHLA 02 cells 48 h post transfection with either shRNA targeting env or CRISPRi targeting HML-2 LTR. (**b**) CHLA 02 cells transfected with CRISPRi + gRNA have reduced aggregate size following reduced expression of HML-2 mRNA at 24 h, 48 h, and 72 h post transfection. (**c**) CHLA 02 cells at 72 h post transfection with either pcDNA or (**d**) with CRISPRi + gRNA. (**e**–**i**) Downregulation of HML-2 results in AT/RT cell death and reduced cell aggregates. (**e**) CHLA 02 and CHLA 04 cells transfected with CRISPRi with or without gRNA targeting HML-2 LTR results in different proportions of viable cells at 96 h post transfection. (**f**) Percent of control (siRNA non-targeting) env transcription following transfection with siRNA targeting HML-2 env in CHLA 02 at 24 h. (**g**) CHLA 02 cell aggregates were reduced in size after 72 h following siRNA transfection targeting HML-2 env. (**h**) CHLA 02 at 72 h post transfection with both non-targeting siRNA and (**i**) cells treated with HML-2 env siRNA. (**j**–**m**) Lentiviral vectors with CRISPRi + gRNA targeting HML-2 and shRNA targeting HML-2 env result in decreased AT/RT viability and a reduction in HML-2 env protein expression. (**j**,**k**) CHLA 02 cytotoxicity at multiple time points post-transduction with lentivirus expressing either a CRISPRi construct with CRISPRi + gRNA targeting HERV-K (HML-2) LTR5_Hs or with CRISPRi without gRNA. (**l**) Percent viable cells 48 h post-transfection shRNA targeting HML-2 env. (**m**) env protein post-transfection as a percent of control transfection (either with CRISPRi and no gRNA or shRNA (−)). Graphs generated with Prism v9. [Error bars represent SEM. Statistics in Supplemental Table S6].

HML-2 transcription was downregulated 48 h post transfection with a construct containing shRNA targeting HERV-K env or CRISPRi and gRNA targeting the HML-2 LTR and led to a significant decrease in HERV-K env transcription (Fig. 5a) and in the size of cellular aggregates (Fig. 5a–d). Due to the importance of cellular adhesion for pluripotency^43–45^, we also analyzed cell viability following CRISPRi transfections and found a significant decrease at 96 h post transfection in CHLA 02 and CHLA 04 (Fig. 5e). Downregulation of HML-2 *env* with siRNA (Table 1) resulted in a significant decrease in *env* transcripts (Fig. 5f) and protein expression (Supplemental Fig. S2) accompanied by a decrease in cellular aggregate size at 72 h post transfection (Fig. 5g–i).

**Table 1:** Sequences of siRNA, shRNA, and gRNAs targeting HML-2 expression.

Reagent	Nucleotide sequence	Alignment to consensus gene or consensus LTR5_Hs
siRNA ENV 1	CUGACGCAGUUAGCUACAAUU	166–184
siRNA ENV 2	GAUUCACUUAUCACAUGGUUU	572–590
gRNA sequence 1	GATAGGGAAAAACCGCCTTAAGGG	563–585
gRNA sequence 2	AAAGCAGTATTGCTGCCCGCAGG	600–620
gRNA sequence 3	TCCTGCCTGTCCCTGGGCAATGG	538–560
gRNA sequence 4	AGTAGATGGAGCATACAATCGGG	498–520
shRNA_469	GGGTATCGTTATCCTCCTATT	469–489
shRNA_866	CAGCTGTTGATAGCGACTTAA	866–886
shRNA_1620	CATGAGCTTAGAACATCGTTT	1620–1640

A significant and time-dependent increase in cytotoxicity occurred at 48 h and 72 h in CHLA 02 cells post transduction with HML-2 targeted CRISPRi lentivirus at a MOI of 0.1 and 0.5 (Fig. 5j,k). Two days following nucleofection, significantly fewer viable cells were observed as measured with propidium iodide staining in the shRNA (env) transfected cells (Fig. 5l). Targeting HML-2 env with shRNA or CRISPRi + gRNA resulted in decreased cell proliferation at 48 h and a significant decrease in env protein (Fig. 5m, Supplemental Fig. S2).

### HML-2 expression induces cell proliferation through the NRAS pathway

**Figure 6:**
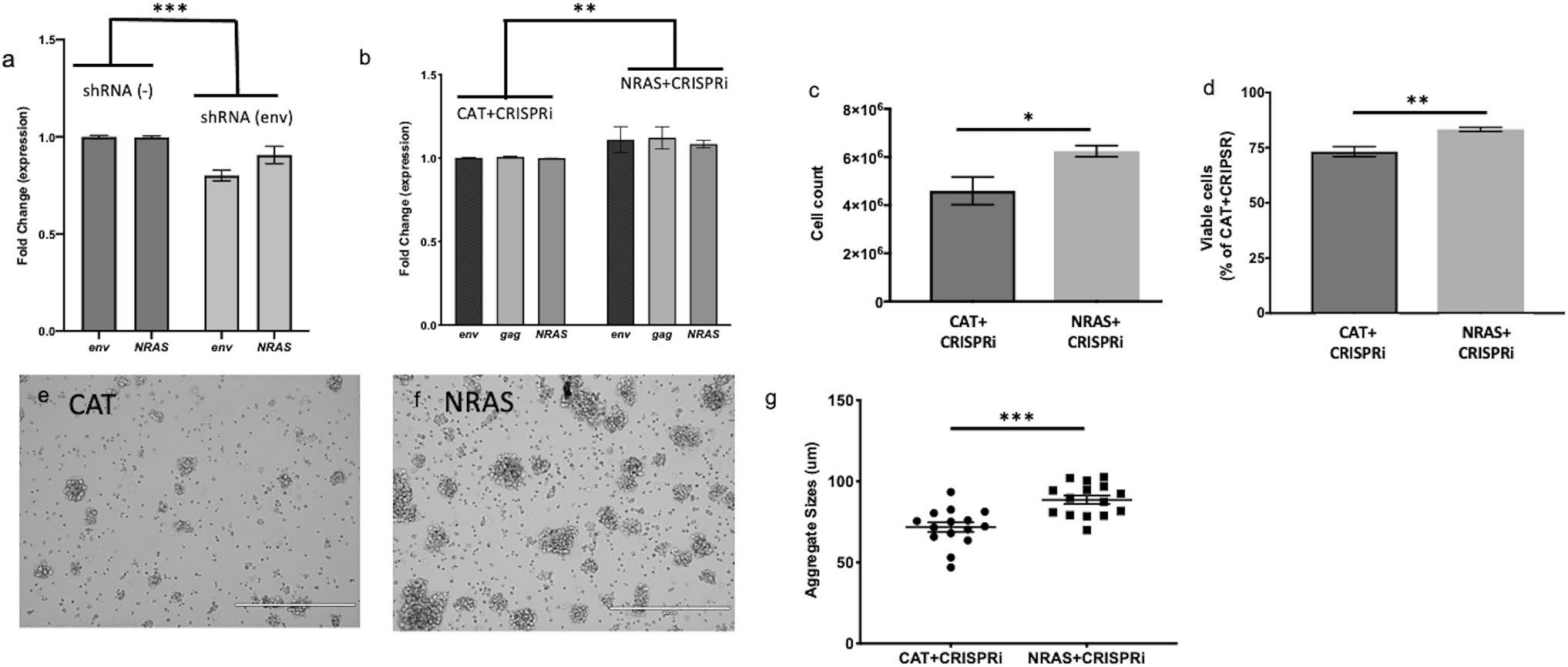
*NRAS* expression is downregulated post HML-2 downregulation, and its overexpression is sufficient to restore cellular proliferation to AT/RT cells. (**a**) Downregulation of HML-2 transcription with shRNA results in a decrease in *NRAS* expression. Transcription level of HML-2 *env* and *NRAS* measured with qPCR 48 h post transfection with shRNA targeting HML-2 *env* in CHLA 02. (**b**–**g**) Co-transfection with *NRAS* overexpression plasmid can overcome the effects of HML-2 downregulation. (**b**) Expression of *env*, *gag*, and *NRAS* transcripts 48 h post transfection in CHLA 04 cells with both CRISPRi + gRNA and *NRAS* plasmids. (**c**) Cell number in CHLA 04 AT/RT cells transfected with CRISPRi + gRNA and either a *CAT* plasmid or an *NRAS* construct and 5 days post-transfection. (**d**) Viable cell percentage 5 days post transfection with CRISPRi and either *CAT* or *NRAS* plasmids. Image of cells transfected with CRISPRi plus HML-2 gRNA and either *CAT* (**e**) or *NRAS* (**f**). (**g**) Quantification of cell aggregate size 5 days post-transfection with CRISPRi + gRNA and either *CAT* or *NRAS* plasmids. Graphs generated with Prism v9. [Error bars represent SEM. Statistics in Supplemental Table S6].

In previous studies of HML-2 downregulation in tumors, a concurrent decrease in N-Ras protein has been observed suggesting possible regulation of *Ras* genes by HML-2^29,30,46^. When CHLA 02 cells were treated with shRNA to HML-2 *env,* there was a significant decrease in *N-Ras* expression alongside HML-2 expression in the CHLA 02 and CHLA 04 cells (Fig. 6a). Forty-eight hours after CHLA 04 transfection with both the CRISPRi construct (+ gRNA targeting HML-2) and with an *NRAS* plasmid, higher transcription of HML-2 and *NRAS* were found by qRT-PCR relative to cells transfected with a pcDNA *CAT* which served as a control (Fig. 6b). This result indicates that NRAS overexpression is sufficient to restore HML-2 transcription. At 5 days post transfection, we observed a significant difference between both the viability and cell number of the *NRAS* and *CAT* transfected cells (Fig. 6c,d). Additionally, we observed the cell aggregates were significantly larger in the cells transfected with both CRISPRi and *NRAS* (Fig. 6e–g), demonstrating a restoration of the cell proliferation of the AT/RT cells.

### The role of C-MYC in HERV-K transcription activation

**Figure 7:**
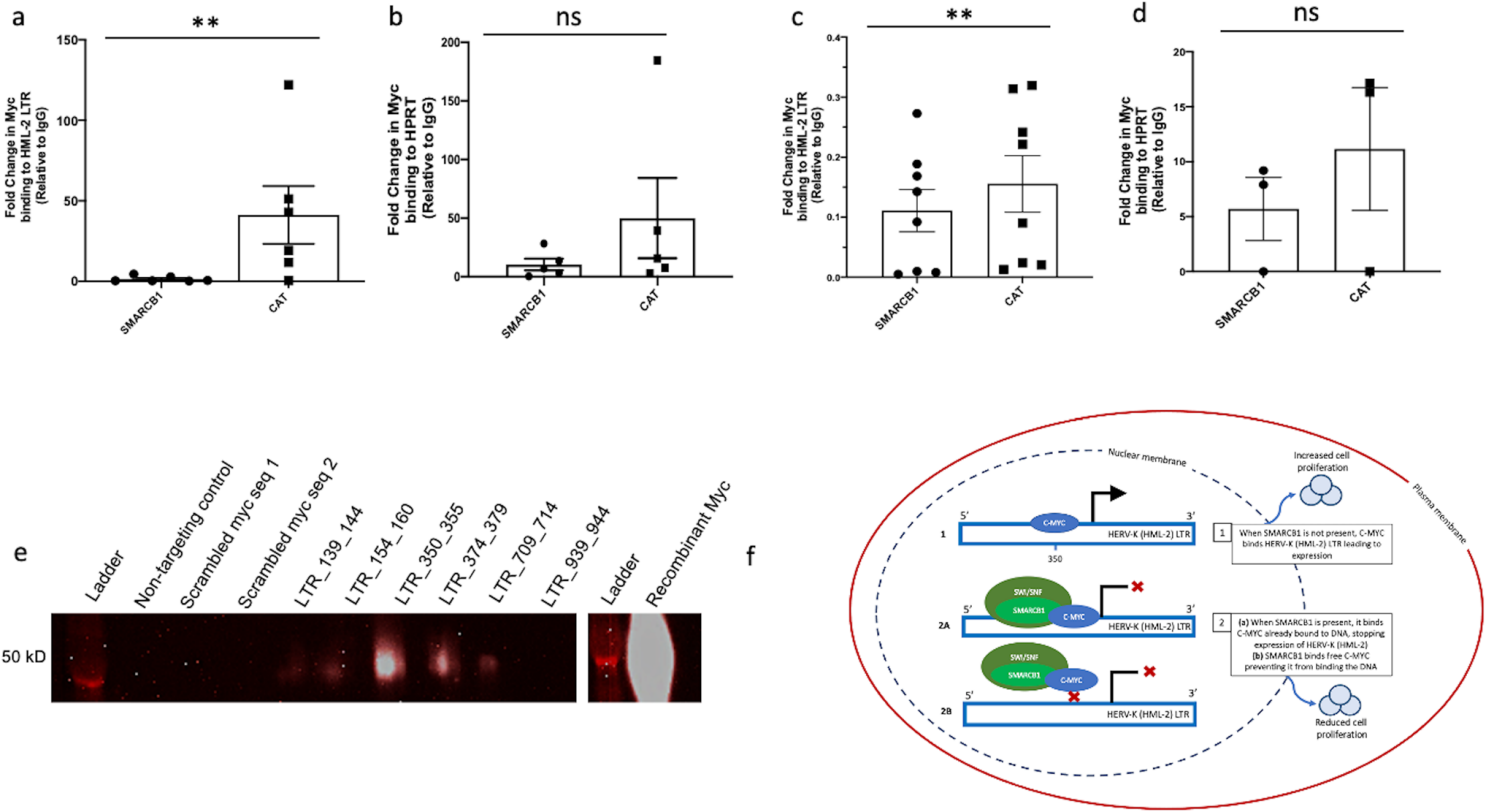
C-MYC binding to the HERV-K (HML-2) LTR. (**a**–**d**) C-MYC binds HML-2 LTR significantly less when *SMARCB1* is expressed. *Panel a:* Relative quantification of HML-2 LTR bound to *C-MYC* transcription factor binding protein immunoprecipitated from either 293 T cells transfected with a *SMARCB1* plasmid or a control (*CAT*) plasmid. (**b**) Relative quantification of housekeeping gene *HPRT* bound to C-MYC post-immunoprecipitation from 293 T cells transfected with either *SMARCB1* or a control (*CAT*) plasmid. (**c**) Relative quantification of C-MYC bound to the HML-2 LTR post immunoprecipitation in CHLA 02 cells transfected with either *SMARCB1* or with *CAT* plasmid. (**d**) Relative quantification of CMYC bound to housekeeping gene *HPRT* post immunoprecipitation in CHLA 02 cells transfected with either *SMARCB1* or with *CAT* plasmid. Percent input is a normalized value with input set to 100% (e.g. % input = 100*2^(input Ct − immunoprecipitated chromatin Ct). Ct is cycle threshold. (**e**) C-MYC binds the HML-2 LTR in vitro. Biotinylated nucleotides with a non-targeting sequence, a scrambled canonical *C-MYC* binding sequence, or varying sequences from the Chr7p22.1a or Chr7p22.1b HML-2 LTR were incubated with recombinant C-MYC protein, washed, and run on an immunoblot to detect specific binding between C-MYC and different sequences. There is a white line between the the final LTR_939_944 and another ladder depicting that a portion of the gel was deleted to simplify the figure. The full gel image can be found in the supplemental information. (**f**) Contains a diagram of the mechanism by which HML-2 expression is regulated in the absence or presence of SMARCB1 protein. (**f1**) shows how HML-2 is actively expressed when C-MYC is bound to the LTR (promoter) when SMARCB1 protein is not expressed, while (**f2**a,b) depict the SMARCB1 mediated inhibition of C-MYC activation of HML-2 transcription. Graphs generated with Prism v9. (**f**) Generated in Microsoft PowerPoint. [Error bars represent SEM. Statistics in Supplemental Table S6].

Many transcription factor binding sites (TFBS) have been reported in the LTR of HERV-K (HML-2) loci; however, not all suggested TFBS have been confirmed^13,14^. In the LTR of Chr7p22.1a and Chr7p221b, both highly expressed loci in AT/RT, there are multiple potential C-MYC binding sites present^47^. Using chromatin immunoprecipitation of crosslinked DNA in 293 T cells, we observed significantly fewer HML-2 LTR5_Hs sequences bound to C-MYC protein when *SMARCB1* was overexpressed (Fig. 7a,b). In the CHLA 02 AT/RT cells, the chromatin immunoprecipitation of C-MYC revealed fewer HERV-K LTR sequences bound to C-MYC in the absence of *SMARCB1* expression (Fig. 7c,d). There was no significant difference between the *SMARCB1* transfected and the control (*CAT*) transfected cells when comparing C-MYC bound to a housekeeping gene, *HPRT*, in both the 293 T and CHLA 02 cells (Fig. 7b,d). We tested binding sites predicted via bioinformatics by PROMO^47^ and other manually identified sites, using a pulldown using biotinylated HERV-K (HML-2) LTR sequences combined with purified C-MYC protein (Supplemental methods). C-MYC protein bound to multiple tested HERV-K sequences and was absent in the non-targeting control as well as the scrambled *C-MYC* sequences tested (Fig. 7e).

## Discussion

We report here that the absence of *SMARCB1* expression in AT/RT leads to aberrant HML-2 env expression in cells which are meant to differentiate along a neuronal lineage. Our study establishes that aberrant regulation of HML-2 expression at the level of chromatin remodeling is sufficient to drive cellular proliferation. In our study of AT/RT, HML-2 activates cell proliferation through the Ras pathway, and the consequences of a targeted decrease in HML-2 expression can be reversed by an overexpression of *NRAS*. These results suggest that improper chromatin remodeling during early neural crest migration and neuronal differentiation provide a unique opportunity for HML-2 env expression to be retained via C-MYC binding and thereby maintain an undifferentiated phenotype. We show that HML-2 env downregulation through transcriptional repression reduces functional env protein, resulting in reduced proliferation and cytotoxicity.

It has been suggested that some HERVs are selectively maintained in the human genome due to their highly regulated expression patterns during development^25,48^. In cells where HML-2 has been dysregulated during key developmental stages, development cannot proceed^25^. Using tissue arrays we found that 95% of tested AT/RT samples expressed HML-2 env. We confirmed expression of a pluripotency gene, *OCT4*, and HML-2 *env* genes in AT/RT cell lines by immunostaining. The cells also expressed neuronal stem cell and neuronal markers suggesting these cells are partially differentiated along a neuronal lineage. Consistent with the cellular heterogeneity and multiple stages of differentiation in AT/RT, HML-2 Env was not uniformly expressed in the tumors. Our results support previous findings in pluripotent stem cells, embryonic stem cells, embryonic carcinoma cells, and many other cancers^10,25,29,30,46,49–67^.

We observed HML-2 Env expression in two AT/RT cell lines in extracellular vesicles by immunocytochemistry (ICC), Immunoblot, and with electron microscopy. The extracellular vesicles containing HML-2 Env could serve as a continuous signal to adjacent cells to maintain pluripotency thereby supporting tumor survival^68^. Further, the expression of proteins related to viral nucleoproteins, nucleocapsids, virions, cell–cell junctions, cadherin cell–cell binding, and cellular adhesion likely contribute to tumor survival and growth. The enrichment of cellular interaction and attachment proteins reflects the morphology of the cells in vitro and affirms the importance of plasma membrane contact between adjacent cells^44^. Cadherins are calcium dependent transmembrane proteins which are vital for cell adhesion^45^, and E-cadherin expression promotes adhesion between adjacent cells^43,69,70^. Cell–cell communication is key in a tumor to adapt to microenvironmental shifts. In AT/RT, extracellular vesicles with HML-2 env combined with the expression of cellular adhesion proteins may enable the tumor to efficiently adapt to its microenvironment.

RNA sequencing showed that there were several active loci from which HML-2 *env* transcripts were produced. This was confirmed by cloning and sequencing the transcripts. All four cell lines expressed transcripts originating from three loci capable of encoding a full length Env protein on Chr19q11, Chr7p22.1a, or Chr7p22.1b. Targeting HML-2 *env* with shRNA has been previously shown to lead to reduced cellular proliferation and decreased metastatic potential of breast cancer in vitro through the inhibition of tumor associated genes like *Ras, p-RSK,* and *p-ERK*^29^. To further investigate the role of HML-2 *env* in AT/RT tumorigenesis, we modulated its expression via siRNA and shRNA and found a significant increase in cytotoxicity and decrease in cellular proliferation. After transfecting CHLA 02 with shRNA targeting HML-2 *env*, we observed a decrease in *NRAS* expression concurrent with the decrease in proliferation. An overexpression of *NRAS* was sufficient to rescue AT/RT cells from the loss of HML-2 env expression. Further, C-MYC binding to the HERV-K LTR is significantly increased in the absence of *SMARCB1* resulting in high HERV-K expression. These findings suggest that HML-2 env plays a role in cellular viability and proliferation.

Our study establishes a new connection between chromatin remodeling and the regulation of endogenous retroviral elements in disease. We demonstrate that the regulation of HML-2 env expression early in development or differentiation is critical to maintenance of pluripotent stem cell identity in AT/RT. When the SWI/SNF chromatin remodeling complex loses a core subunit, *SMARCB1*, during development, the aberrant activation of human endogenous retroviral genes can contribute to the maintenance of stem cell features in cells which were meant to differentiate during CNS development. In addition to aberrant HML-2 transcription in the absence of SMARCB1, the transcription factor binding protein C-MYC binds the HML-2 LTR more frequently and upregulates its transcription. This study introduces a new mechanism of endogenous virus-mediated tumorigenesis. Identification of HML-2 expression as a marker of AT/RT following the loss of SMARCB1 is a primary step in establishing the role of endogenous retroelements in this central nervous system tumor development. Based on the findings in our study, HML-2 env expression may have a role as a marker for diagnosis of AT/RT in conjunction with SMARCB1. Further studies are needed to determine if HML-2 expression may be of prognostic significance and if it may be a therapeutic target.

## Methods

### Cell culture

CHLA AT/RT cell lines and 293 T cells were purchased from ATCC and maintained as recommended in DMEM:F12 with 20 ng/mL human recombinant basic EGF, 20 ng/mL human recombinant basic FGF, and B-27 supplement to a final concentration of 2%. iPSCs, NSCs, and neurons were cultured as documented in^51^. Cell line information from ATCC is as follows: CHLA 02 (CRL-3020), CHLA 04 (CRL-3036), CHLA 05 (CRL-3037), and CHLA 06 (CRL-3038).

### Immunofluorescence

The AT/RT cell lines CHLA 02, CHLA 04, CHLA 05, and CHLA 06 all grow in suspension; therefore, the cells were attached to the bottom of the plate with Matrigel for staining and grew overnight at 37 °C. Next, they were fixed with 4% paraformaldehyde (PFA) for 10 min. The cells were washed with 1X phosphate buffered saline (PBS) 2 × for 5 min each and then permeated with PBS with 0.05% triton X 100 (PBST) for 10 min. Cells were incubated with a blocking solution of 5% donkey or goat serum in PBS for 1 h and then washed with PBS 3 × for 5 min each and primary antibodies diluted in blocking solution were applied to the samples. After an overnight incubation at 4 °C, the cells were washed 3 × for 10 min each with PBS and then incubated with the appropriate secondary antibodies at 1:400 dilution for 1 h. Following the secondary antibody, the cells were treated with 4′,6-diamidino-2-phenylindole (DAPI) at 1:10,000 and washed 2 × with PBS before imaging with a fluorescent Zeiss confocal microscope (LSM510) in Fig. 2, images were acquired with Zeiss LSM 5 Image Browser and processed in Microsoft PowerPoint for figure creation. For Fig. 1 immunoflourescence images, an EVOS fluorescence microscope (AMG) was used. Images were acquired with the native software installed on the EVOS microscope and processed in Microsoft PowerPoint. Primary antibodies: AntiCD98 antibody ab108300; anti-Tubulin β3 801213 (Bio-legend), Anti-Oct4 antibody AB3209 (Millipore sigma), Anti-nestin Mab 5326 (Millipore), Anti-Pax6 NBP1 51622 (Novus biologicals). Secondary antibodies: Goat Anti-Rabbit IgG (Alexa Fluor 488, ab150077), Goat Anti-Mouse IgG (Alexa Fluor 594 ab150116), Goat-anti Rabbit IgG (Alexa Fluor 594, ab150080), Goat anti-mouse IgG (Alexa Fluor 488, ab150113) used at 1:500 dilution. Validation of the polyclonal antibody (PAb) is shown in Supplemental Fig. S3. The peptides used to make HERV-K env polyclonal antibody were as follows:

QRKAPPRRRRHRNRC (HERV-K env amino acid position: 8–21), CSDLTESLDKHKHKK (env amino acid position: 294–307), and CSKRKGGNVGKSKRD (env amino acid position: 680–693).

### Electron microscopy (EM)

CHLA 02 cells were pelleted at 2,000 RPM for 5 min and 3 million cells were resuspended in 700 ul of media. 4% room temperature glutaraldehyde was made in a 1% cacodylate buffer. 700 ul of glutaraldehyde solution was added to cell/media mixture and gently mixed in Eppendorf tube. One drop of 22% albumin was added to tube and the sample was incubated at room temperature for 30 min. Following room temperature incubation, sample was stored at 4 °C until tubes are ready to be further processed for.

EM. When ready for silver enhancement, cells were washed with deionized water thoroughly (5 × 5 min). Silver enhancement was then performed (under safety light) and cells were washed in water (at least 5 times over 10 min). The sample was washed in 0.1 M phosphate buffer, then with 0.2% OsO_4_ in 0.1 M phosphate buffer for an additional 30 min. Next, dehydration was performed in ETOH and sample was embedded in resin. Samples were then cut with an ultramicrotome into ultrathin sections and are placed on a copper metal grid.

### Transfections

The CHLA cell lines were transfected using the P3 nucleofector kit (Lonza, catalog no. V4XP-3012) and the CA -137 program at 1 ug of plasmid per million cells. After transfection, the cells were re-suspended in at least 4 mL of media. Sequences for shRNA, siRNA, and gRNAs are included in Table 1. The 293 T cells were transfected with Lipofectamine 3000 following manufacturer’s recommendations using 5 ug of plasmid DNA per million cells. SMARCB1 lentiviral plasmid was obtained from Addgene. The pcDNA *CAT* plasmid contained a bacterial acetylcholine transferase. The *CAT* construct was used to control for effects of transfection as a plasmid of similar size to the SMARCB1 plasmid^71^. pDONR223_SMARCB1_WT was a gift from Jesse Boehm & William Hahn & David Root (Addgene plasmid # 81791; http://n2t.net/addgene:81791; RRID:Addgene_81791)^72^. pHAGE-NRAS was a gift from Gordon Mills & Kenneth Scott (Addgene plasmid # 116767; http://n2t.net/addgene:116767; RRID:Addgene_116767). A CRISPRi construct was used, comprised of a plasmid with a CRISPR-dCas9 (dead Cas9), four fused Sin3 repressive interacting domains (SID), along with four gRNAs targeting HML-2 LTR5_Hs (SID4X) (Table 1).

SID is a chromatin remodeling protein^73,74^ that prevents transcriptional machinery from accessing the DNA resulting in decreased expression of the targeted gene. As a control, cells were transfected with pcDNA and the transfection related toxicity was normalized accordingly in the CRISPRi transfected cells.

### Immunoblotting

Immunoblotting was performed as done previously^26^ with the exception of the transfer which was performed with the iBlot 2 (ThermoFisher, IB24001). In addition, a new polyclonal antibody to detect HML-2 SU (surface unit) Env was used (additional information and antibody validation can be found in Supplemental Fig. S2 and Supplemental methods.) All images were quantified with ImageJ. The HML-2 Env antibody used in Supplemental Fig. S2 targets the transmembrane portion of the protein. The transmembrane antibody was made using the immunogen CSKRKGGNVGKSKRD, used at a concentration of 1:1000, and was first mentioned in a previous manuscript^75^.

### Immunohistochemistry

Immunohistochemistry detected cells with Human endogenous retroviral Env expression primary antibody (Austral Biologicals, HERM 1855) at a 1:500 concentration within formalin fixed paraffin embedded tissue samples. Further detail can be found in Supplemental methods.

### Enrichment of extracellular vesicles using nanotrap particles

Nanotrap particles, NT82 particles (#CN2010) and NT80 particles (#CN1030) (Ceres Nanosciences, Inc.) have been previously shown to be effective in the enrichment of extracellular vesicles from cell culture supernatant and patient biofluids^76,77^. Equal volumes of these two particles were combined with 1X PBS without calcium or magnesium to create a 30% slurry. For capture of extracellular vesicles (EVs) from cell culture supernatant, 30 µL of the 30% slurry was added to 1 mL of cell-free supernatant and rotated at 4 °C overnight. The following day, nanoparticles were pelleted at 10,000×*g* for 10 min at room temperature.

The resulting pellets were washed once with 1X PBS without calcium and magnesium and resuspended in 20 µL of Tris–Glycine SDS sample buffer with 10% 2-Mercaptoethanol. Samples were heated at 95 °C for 15 min with vortexing and loaded onto a 4–20% Tris/Glycine gel (Invitrogen). Gels were run at 100 V and transferred at 20 V for 7 min using iBlot 2 Gel Transfer Device. Membranes were blocked in 5% milk in PBST for 2 h at 4 °C. then incubated at 4 °C overnight in PBS-T with HML-2 envelope polyclonal antibody against the transmembrane protein (see Immunoblot section in Supplementary methods).

### RT-PCR, PCR, and sequencing of env transcripts

RNA was isolated from iPSC, NSC, neuronal, and AT/RT cell lines with a Qiagen RNeasy kit (Qiagen, 74104) and reverse transcribed with the SuperScript First-Strand Synthesis RT-PCR kit (ThermoFisher Scientific, 11904018). Polymerase chain reactions were used to amplify HML-2 envelope transcripts with primers which target the full length *env* transcript (Table 1). Q5 high fidelity 2X master mix was used for the PCR with the following cycling conditions: 98 °C for 90 s, [98 °C 10 s, 55 °C 20 s, 72 °C 4 min] repeat 34 times, final extension at 72 °C for 6 min, 4 °C forever. The PCR primers were used to amplify the full length env gene as following: forward primer 5’- cccactagacatttgaagttctaca-3’, and reverse primer 5’- ggagtctcctatgtctacttcttt-3’. One primer aligned to the 3’ LTR of the HML-2 element and the other primer was positioned 5’ (upstream) of the start of the env gene. For the AT/RT cells, we cloned products into Topo-TA vectors (ThermoFisher Scientific, K203001) and sent them for Sanger sequencing to determine which env loci were actively transcribed in the samples. For the iPSCs, NSCs, and neurons, PCR products were run in a 2% agarose gel with GelStar Nucleic Acid Gel stain (Lonza, 50535) and the gel was imaged using Flourchem Protein Simple imager.

### Alamar Blue viability assay

To distinguish the effect of HML-2 downregulation on AT/RT viability the Alamar Blue cell viability reagent (Thermofisher, DAL1100) was used. The fluorescence of the cell media resulting from the reduction of the Resazurin to resorufin indicated the percent of viable cells. Viability was calculated by comparing the fluorescence of each treatment concentration divided by the viability of the control treatment/ transfection.

Each experiment was performed with at least three technical replicates and three biological replicates.

Plates were read on the FlexStation 3 using Softmax Pro with the ‘blue fluorescence’ at 590 nm viability assay setting.

### Lentiviral production

Lipofectamine 3000 (Invitrogen L3000008) kit was used to transfect 293 T cells with either a lentiviral construct containing four repressive Sin3 domains, dead Cas9, and four gRNAs designed for HERVK LTR5_Hs or one without gRNA. The aforementioned CRISPRi construct was incorporated into a lentiviral vector, both with and without gRNA targeting the LTR5_Hs, and AT/RT cells were transduced with the lentivirus at MOIs of 0.1 and 0.5. Manufacturer’s guidelines were followed for the transfection. Further information can be found in the Supplementary methods.

### RNA sequencing

Libraries were both prepared and sequenced at NYGC (www.nygenome.org). For library preparation, the Illumina TruSeq Stranded Total RNA protocol was used (Illumina, San Diego, CA). Per sequencing, each library was paired-end sequenced (125 bp) for a target depth of 40 million reads (HiSeq 2500 Illumina); providing for a pair of .fastq files per library post CASAVA deplexing accessible at NCBI as GSE124210.

For further detail regarding analysis, please see Supplemental methods.

### Chromatin immunoprecipitation (ChIP) and qRT-PCR

Five million cells were collected for each ChIP experiment from either pcDNA *CAT* transfected cells or cells transfected with *SMARCB1*. Cells were fixed with 1% PFA in fresh media and crosslinking was stopped by the addition of 1.25 M glycine to a final concentration of 0.125 M. Cell pellets were then snap frozen for storage at − 80 °C until ChIP was performed. Specific buffers and additional information about the procedure can be found in the Supplemental methods. To obtain the ratio of specific sequences pulled down during ChIP, semi-quantitative PCR was performed using primers that spanned the HML-2 transcription start site (TSS): LTR Forward (5'-GTT TGT CTG CTG ACC CTC TC-3') and Reverse (5’-AGC CTC TGA GTT CCC TTA GT-3’); qPCR was also performed using primers for an unrelated genomic region (hypoxanthine phosphoribosyltransferase 1; HPRT1), Forward (5’-GCT GAC CTG CTG GAT TAC AT-3’) and Reverse (5’-GGT TTG CAG AGA TTC AAA GAA-3’). Results are shown as percent of input chromatin, calculated using the formula % input = 100*2^(Ct^_[input]_ − ^Ct^_[IP]_). Antibodies used for ChIP: Go-ChIP grade purified Anti-RNA polymerase II antibody, 904,004, Biolegend. Mouse Mab IgG XP (R) isotype control antibody, Cell Signaling, 3900S. *Ini1* antibody (A-5): sc-166165 (*Ini1* is another name for *SMARCB1*), Santa Cruz.

### Mass spectrometry

#### In-gel digestion of PAGE fractionated cell lysates

The entire lane was excised from the PAGE gel, sectioned into 16 approximately equivalent sections, transferred to 1.5 mL microfuge tubes and subjected to a modified in-gel digestion protocol^78^. Briefly, excised bands were destained by adding 0.5 mL 100 mM NH_4_HCO_3_:CH_3_OH (50:50, v/v) and incubated in a thermomixer (800 RPM, 37 °C) for 60 min. Solvent decanted and discarded, process repeated as necessary. Gel-immobilized proteins were reduced by adding 0.5 mL 100 mM NH_4_HCO_3_, 5 mM DTT, incubated in a thermomixer (800 RPM, 50 °C) for 30 min, and allowed to cool to RT. Reduced proteins were carboxamidomethylated by buffer exchange into 0.5 mL 100 mM NH_4_HCO_3_, 12.5 mM IAM and incubated in the dark for 30 min at RT. Gel bands were washed extensively by sequential buffer exchange using 0.5 mL aliquots of 100 mM NH_4_HCO_3_, 100 mM NH_4_HCO_3_:CH_3_CN (50:50, v/v; 0.5 mL), and neat CH_3_CN (0.25 mL). In each instance, the gel bands were incubated in a thermomixer (800 RPM, 37 °C) and the solvent decanted and discarded.

Gel bands were rehydrated using 25 uL 100 mM NH_4_HCO_3_ containing sequencing grade trypsin (Promega Corp.) at 10 ng/uL (0.4 pmol/uL). After 30 min, additional 100 mM NH_4_HCO_3_ was added to completely cover the gel bands (approx. 200 uL total vol.). Samples were incubated in a thermomixer (800 RPM, 37 °C) for 18 h.

Peptides were recovered by decanting the supernatant to a new 1.5 mL microfuge tube and extracting the gel bands with an equivalent volume of H_2_O:CH_3_CN:CF_3_CO_2_H (20:80:0.25; v/v/v) and sonicating for 30 min.

Extracts were combined with original supernatants and lyophilized to dryness using a vacuum centrifuge.

All samples were desalted by solid phase extraction using TARGA C18 microspin columns according to manufacturer’s instructions (The Nest Group). Desalted samples were resolubilized in 10 μL 1% CF_3_CO_2_H transferred to an autosampler vial.

#### Liquid chromatography-mass spectrometry

Nanoflow LC–MS/MS was performed using an UltiMate 3000 RSLCnano UHPLC directly coupled to an Orbitrap Fusion Lumos mass spectrometer (Thermo Fisher Scientific, Inc., San Jose, CA) similarly to previous studies^35^. An integrated autosampler was used to load samples at 5 μL/min onto a nanoViper trap column (75 mm × 20 mm, Acclaim PepMap 100 C18 resin, 3 μm particle size, 100 Å pore size, PN: 164535). Reversed phase HPLC was performed using an EasySpray C18 column (75 mm ID × 750 mm; Acclaim PepMap 100 C18 resin, 2 μm particle size, 100 Å pore size, PN: ES805). Mobile phases were Buffer A: H_2_O: (CH_3_)_2_SO: HCO_2_H (95:5:0.1; v/v/v) and Buffer B: CH_3_CN: (CH_3_)_2_SO: HCO_2_H (95:5:0.1; v/v/v). After 5 min of isocratic flow (200 nL/min, 2%B) a linear gradient from 2 to 35%B was developed over 66 min, 35–80% B over 4 min, and isocratic flow at 80%B for 10 min. LC–MS/MS experiments were performed using data dependent acquisition with dynamic exclusion enabled (exclusion width =  + /− 10 ppm, repeat count = 2, repeat duration = 15 s, exclusion duration 22.5 s). MS1 scans (scan range 400–1600) were acquired in the Orbitrap mass analyzer (resolution 120,000 at m/z 400), AGC Target = 5.0 × 10^5^, max. injection time = 50 ms).

Charge state exclusion enabled (1 + , ≥ 5 +). Based on relative intensity (threshold = 1.0 × 10^4^), up to 20 ions from each survey scan were sequentially isolated and fragmented by HCD (quadrupole isolation width = 0.7 Da, normalized collision energy = 35%, AGC Target = 1.0 × 10^4^).

Raw MS files were processed using Proteome Discoverer 2.2 software. MS/MS spectra were searched against a custom database constructed from the SwissProt portion of the UniProt database (release 2018_7). The database included all HUMAN sequences in SwissProt (curated) and was augmented with common reagent protein sequences (ASPN_PSEFR, CLOS_CLOHI, CTRA_BOVIN, CTRB_BOVIN, CTRC_BOVIN, GST26_SCHJA, LYSC_PSEAE, PNGF_ELIMR, SAV_STRAV, SPA_STAA8, SPA_STAAU, SPG1_STRSG, SPG2_STRSG, and TRYP_PIG).

Processing workflow parameters were: enzyme = trypsin, maximum missed cleavages = 2, fixed modifications = carbamidomethyl-Cys (C), variable modifications = N-acetyl (Protein), pyro-Glu (Q), and oxidation (M), precursor ion mass tolerance = 10 ppm, product ion mass tolerance = 0.6 Da, mass values = monoisotopic. Consensus workflow parameters: use only high confidence peptide, apply strict parsimony, 1% FDR, and 2 peptide IDs minimum.

### Statistics

Prism ‘analyze’ tool used for all t-tests and ANOVA calculations. For all biological assays, at least 3 technical replicates and 3 biological replicates were measured and used for calculation of significance unless otherwise noted. P < 0.05 was considered significant for all statistical tests performed and generally * denotes P < 0.05, ** denotes P < 0.01, *** denotes P < 0.001, and **** denotes P < 0.0001. See statistics table for further information (Supplemental Table S6).

## Supplementary Information

Supplementary Figure 1.

Supplementary Figure 2.

Supplementary Figure 3.

Supplementary Information 1.
