# Supplementary figures and images for: SMARCB1 deletion in atypical teratoid rhabdoid tumors results in human endogenous retrovirus K (HML-2) expression
Source: Sci Rep. 2021 Jun 18;11:12893. doi: 10.1038/s41598-021-92223-x (PMC8213802; doi:10.1038/s41598-021-92223-x)

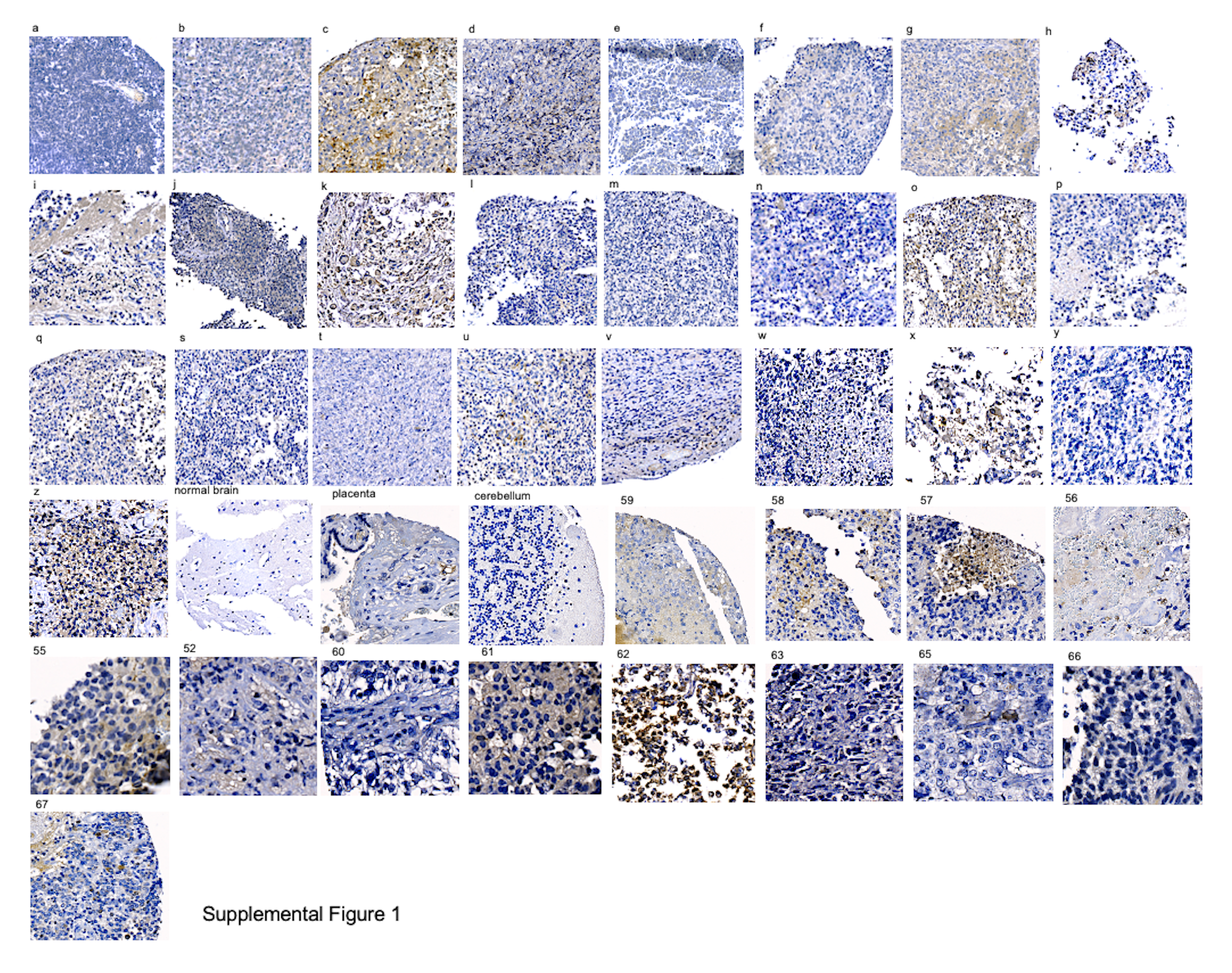

Supplement: Supplementary file 1 — Supplementary Figure 1. [file 41598_2021_92223_MOESM1_ESM.tiff]

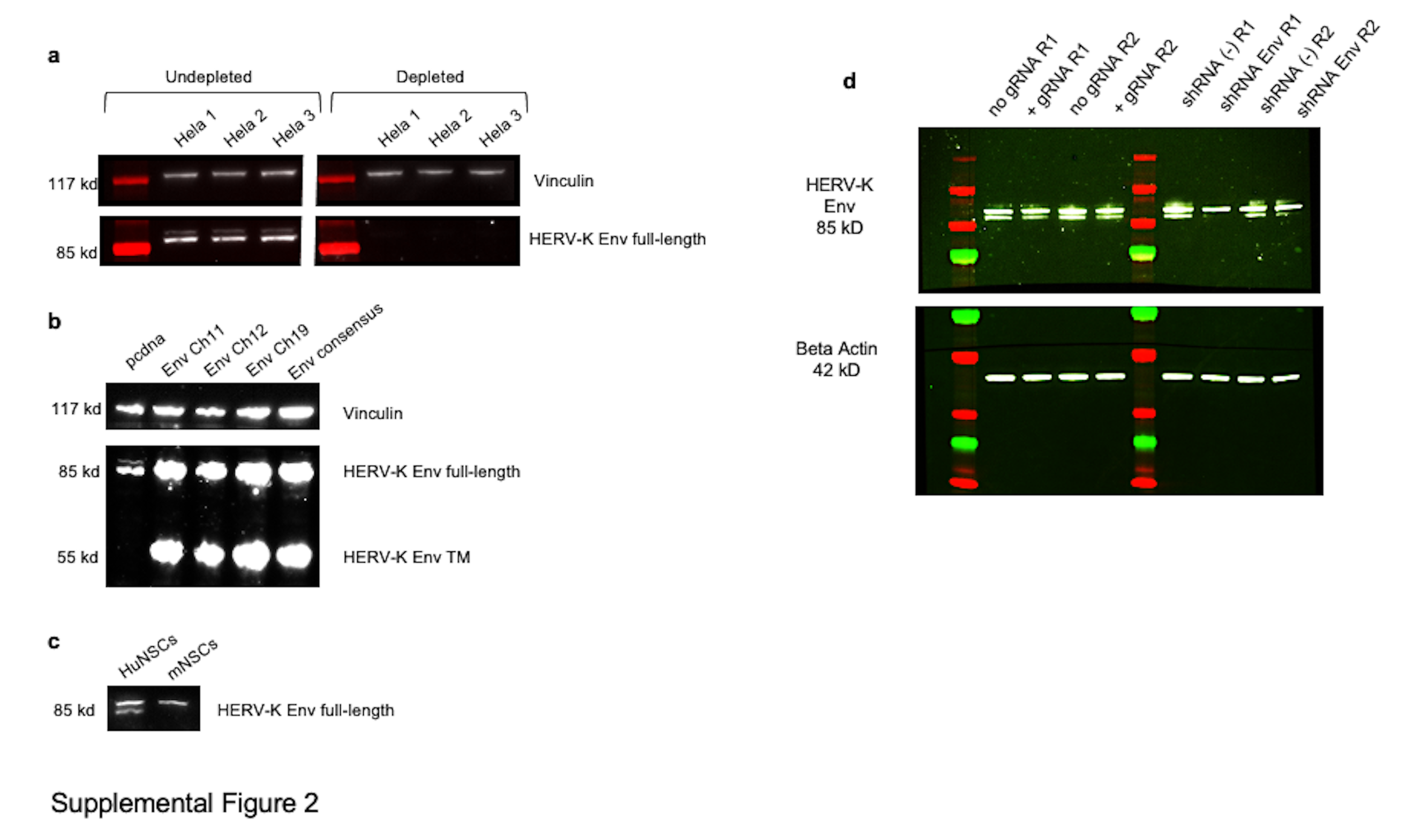

Supplement: Supplementary file 2 — Supplementary Figure 2. [file 41598_2021_92223_MOESM2_ESM.tiff]

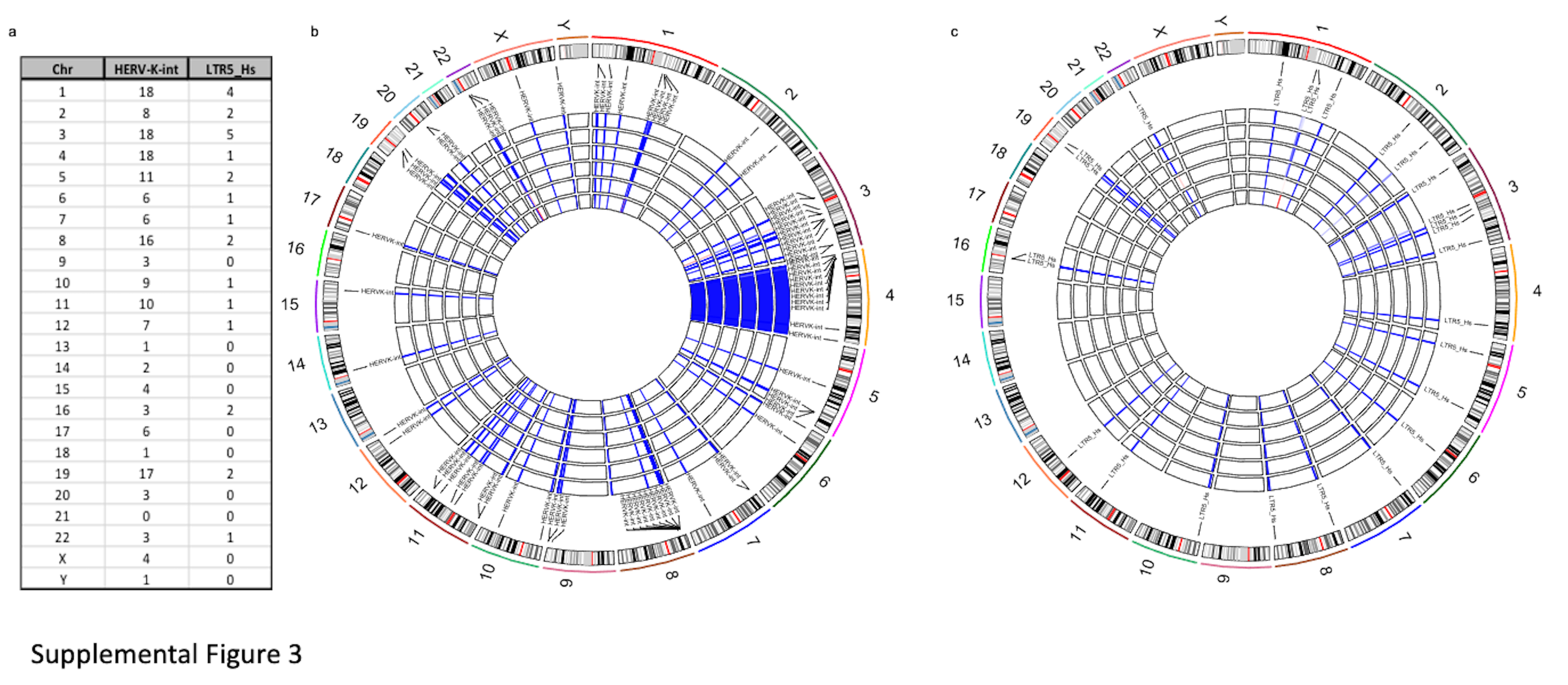

Supplement: Supplementary file 3 — Supplementary Figure 3. [file 41598_2021_92223_MOESM3_ESM.tiff]
