# Supplementary material for: SMARCB1 deletion in atypical teratoid rhabdoid tumors results in human endogenous retrovirus K (HML-2) expression
Source: Sci Rep. 2021 Jun 18;11:12893. doi: 10.1038/s41598-021-92223-x (PMC8213802; doi:10.1038/s41598-021-92223-x)
Supplement: Supplementary file 4 — Supplementary Information 1. [file 41598_2021_92223_MOESM4_ESM.docx]

**SMARCB1 deletion in atypical teratoid rhabdoid tumors results in human endogenous retrovirus K (HML-2) expression**

Tara T Doucet-O’Hare Ph.D.^1^; Brianna L. DiSanza B.A.^1^; Catherine DeMarino, Ph.D.^1^; Abigail L. Atkinson B.S.^1^; Jared S. Rosenblum M.D.^3^; Lisa J. Henderson Ph.D.^1^; Kory Johnson Ph.D.^2^; Jeffrey Kowalak Ph.D.^4^; Marta Garcia-Montojo, Ph.D.^1^; Sariah J Allen Ph.D.^5^; Brent A. Orr M.D. Ph.D.^5^; Mariarita Santi M.D. Ph.D.^6^; Tongguang Wang M.D, Ph.D.^7^; Saeed Fathi B.S.^1^; Myoung Hwa Lee Ph.D.^1^; Kevon Sampson M.S.^1^; Wenxue Li Ph.D.^1^; Zhengping Zhuang M.D. Ph.D.^3^, and Avindra Nath, M.D.^1^*

^1^Section of Infection of the Nervous System, National Institute of Neurological Disorders and Stroke (NINDS), National Institutes of Health (NIH), Bethesda, MD ^2^Bioinformatics Unit; ^3^Neuro-oncology branch, National Cancer Institute (NCI); ^4^Clinical Proteomics Unit; ^5^Department of Anatomic Pathology, St. Jude’s Children’s Research Hospital, Memphis, TN; ^6^Department of Pathology, Children’s Hospital of Philadelphia, Perelman School of Medicine, University of Pennsylvania Philadelphia, PA; ^7^Cell Differentiation Unit; Translational Neuroscience Center, NINDS, NIH, Bethesda, MD.

Address correspondence to:

Avindra Nath MD, Bldg 10; Room 7C-103; 10 Center Drive, Bethesda, MD 20892; Tele: 301-496-1561; e-mail: natha@ninds.nih.gov

**Supplemental Figure 1: AT/RT immunohistochemistry for HERV-K expression.** *Panels A-Z, 59-67:* HERV-K Env monoclonal antibody immunostaining of patient tissue from AT/RT tumors, normal brain, placenta and cerebrum.

**Supplemental figure 2: Verification of HERV-K Env antibody specificity.** *Panel A-D:* HERV-K (HML-2) Env Antibody Validation. *Panel A:* Representative blot of 3 different lysates of Hela cells stained with HERV-K (HML-2) Env antibody (undepleted) or with HERV-K Env antibody incubated overnight with recombinant HERV-K (HML-2) Env (depleted). *Panel B*: Representative Western blot of 5 different lysates of Hela cells transfected with an empty vector (pcdna) or with plasmids vectors which had different sequences of HERV-K Env: locus Chr11, locus Chr12, locus Chr19 and consensus sequence stained with the HERV-K Env antibody. *Panel C*: Immunoblot of 2 lysates, human neural stem cells (HuNSCs) and mouse neural stem cells (mNSCs), stained with the HERV-K Env antibody. *Panel D*: Representative immunoblot of CHLA 02 AT/RT cells transfected with (1) lentiviral plasmid with SID4X with gRNA targeting HERV-K LTR5_Hs or (2) without gRNA, (3) CHLA 02 AT/RT cells transfected with an AAV vector with shRNA targeting HERV-K Env or (4) lacking shRNA. The lower band of HERV-K Env is the main band affected by downregulation mechanisms (e.g. transcriptional downregulation with SID4X or with shRNA targeting Env). The bottom half of the immunoblots display the expression of Beta Actin used as a loading control. The blots in this supplemental figure A,B, and C were cropped for clarity (as denoted by the white lines between blots), the full blot images are below.

**Supplemental Figure 3:** **RNA Sequencing analysis of HERV-K (HML-2) Transcription in AT/RT Cell Lines.** *Panel A*: Table showing quantity of HERV-K internal coding genes and LTR5_Hs loci expressed on each chromosome from the TEtranscripts analysis. *Panel B*: Graphical depiction of HERV-K-internal gene expression from TEtranscripts analysis of RNA-sequencing data. The inner rings represent the following samples going from outermost to innermost: CHLA 02, CHLA 04 technical replicate 1, CHLA 04 technical replicate 2, CHLA 05, CHLA 06 technical replicate 1, and CHLA06 technical replicate 2. The technical replicates show a high level of concordance from the analysis supporting the validity of the data. Blue and red lines correspond to the level of expression of the HERVK internal genes labeled at that location (red denotes higher expression, blue denotes less). *Panel C:* Graphical depiction of LTR5_Hs expression from TEtranscripts analysis of RNA-sequencing data. The inner rings represent the following samples going from outermost to innermost: CHLA02, CHLA04 technical replicate 1, CHLA04 technical replicate 2, CHLA05, CHLA06 technical replicate 1, and CHLA06 technical replicate 2. The technical replicates show a high level of concordance from the analysis supporting the validity of the data. Blue and red lines correspond to the level of expression of the LTR5_Hs labeled at that location (red denotes higher expression, blue denotes less).


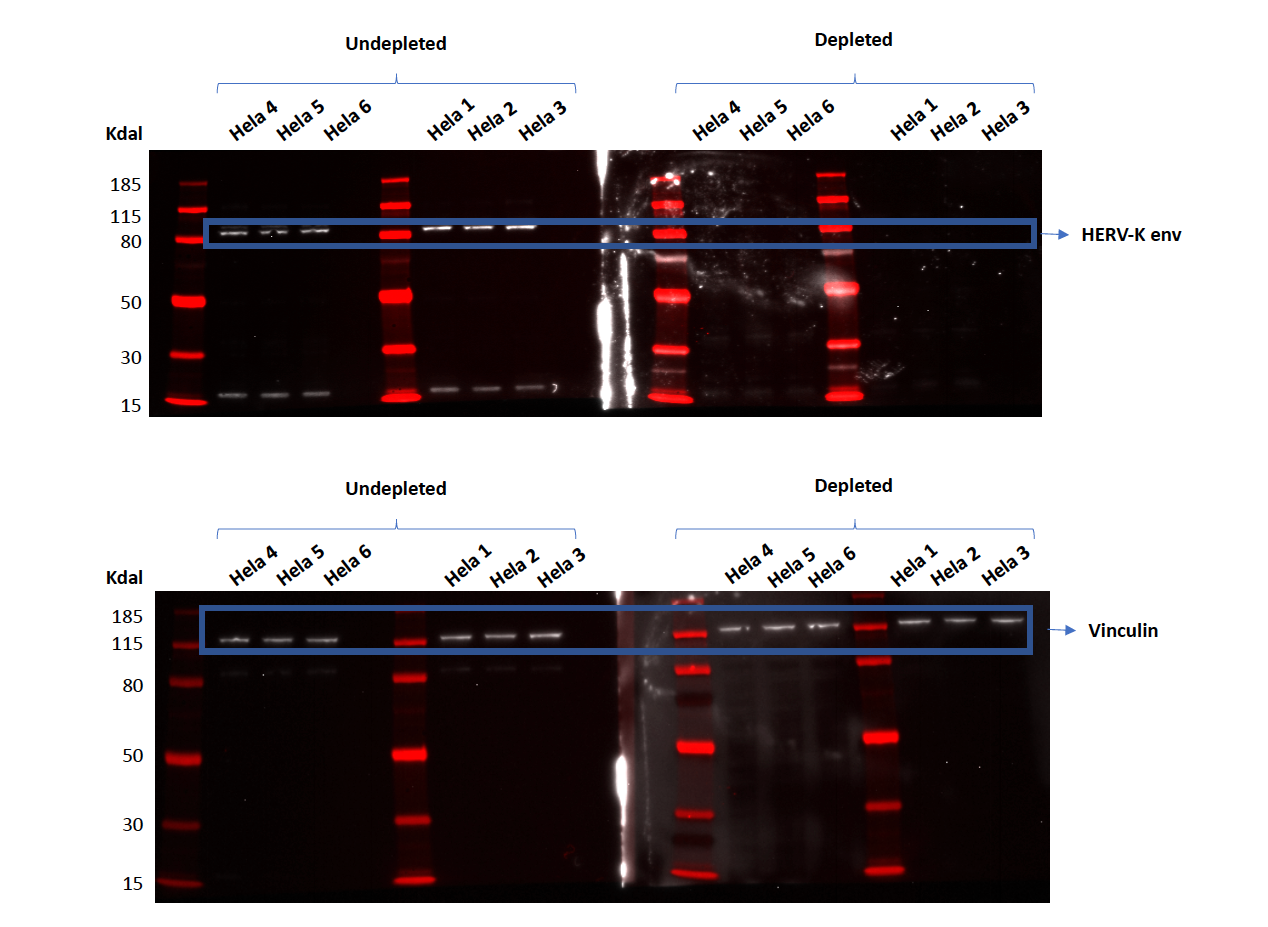


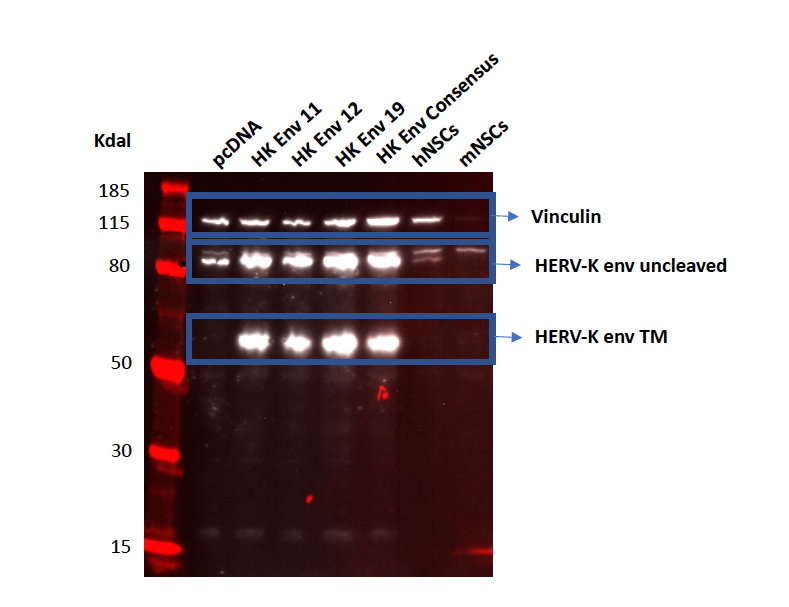


**Supplemental Table 1:** Clinical characteristics, SMARCB1 and HML-2 expression in AT/RT tumors**.**

| **Supplemental Table 1** |  |  |  |  |  | | | |
| --- | --- | --- | --- | --- | --- | --- | --- | --- |
| **UID** | **Identifier** | **Age** | **Sex** | **Site** | **Ancillary testing** | **Recurrence/ Dissemination** | **Additional treatment** | **env**  **Intensity** |
| PC13-  43452 | 52 | 29 (mo) | M | CPA | IHC negative for *SMARCB1*/ gene analysis | Yes, 4 months post-surgery | Some chemotherapy | + |
| PC13-  43455 | 55 | 57 (mo) | M | Cerebellum | IHC negative for *SMARCB1*/ gene analysis | No | Radiation/  Chemotherapy | ++ |
| PC13-  43456 | 56 | 40 (third recurrence of tumor, patient 57) | F | Right cerebrum | IHC negative for *SMARCB1*/ gene analysis | Yes | Radiation/  Chemotherapy | + |
| PC13-  43457 | 57 | 8 (mo) |  |  |  |  |  | ++ |
| PC13-  43458 | 58 | 29 (mo) | M | Right temp/parietal | IHC negative for *SMARCB1*/ gene analysis | Yes, 7 months post surgery | not available | ++ |
| PC13-  43459 | 59 | 53 (mo) | M | Left temporal | H&E morphology/ IHC (EMA, vimentin, SMA, Synaptophysin, GFAP) | Yes, 9 months post surgery | not available | + |
| PC13-  43460 | 60 | 24 (mo) | F | Thalamus | H&E morphology/ IHC (EMA, vimentin, SMA, Synaptophysin, GFAP) | not available | not available | +/- |
| PC13-  43461 | 61 | 11 (mo) | M | Cerebellum | H&E morphology/ IHC (EMA, vimentin, SMA, Synaptophysin, GFAP) | not available | not available | ++ |
| PC13-  43462 | 62 | 45 (mo) | M | Frontal lobe, RT | H&E morphology/ IHC (EMA, vimentin, SMA, Synaptophysin, GFAP)  H&E morphology/ IHC (EMA, vimentin, SMA, Synaptophysin, GFAP) | not available | not available | +++ |
| PC13-  43463 | 63 | 45 (mo,  second tumor in patient 62) | M | Frontal lobe, RT |  | not available | not available | +/- |
| PC13-  43465 | 65 | 45 (mo) | M | CPA/ cerebellum | H&E morphology/ IHC (EMA, vimentin, SMA, Synaptophysin, GFAP) | Yes, 2 years post-surgery | not available | + |
| PC13-  43466 | 66 | 13 (mo) | M | Cerebellum | H&E morphology/ IHC (EMA, vimentin, SMA, Synaptophysin, GFAP) | Yes | not available | +/- |
| PC13-  43467 | 67 | 61 (mo) | M | Frontal lobe, LT | H&E morphology/ IHC (EMA, vimentin, SMA, Synaptophysin, GFAP) | Yes, 5 years post surgery | Radiation/  Chemotherapy | + |
| ATRT1 | A | 7 (mo) | F | Posterior fossa | IHC negative for *SMARCB1* | not available | not available | +/- |
| ATRT2 | B | 3 (mo) | M | Right front parietal | IHC negative for *SMARCB1* | not available | not available | ++ |
| ATRT3 | C | 5 (mo) | F | Right atrial LV | IHC negative for *SMARCB1* | not available | not available | +++ |
| non-ATRT1 | E | 8 (mo) | M | Left frontal lobe | IHC positive for *SMARCB1* | not available | not available | - |
| ATRT4 | F | 2 (mo) | M | Cerebellum | IHC negative for *SMARCB1* | not available | not available | + |
| ATRT5_A | G | 2 (mo) | F | Cerebrum | IHC negative for *SMARCB1* | not available | not available | ++ |
| ATRT5_B | T | 5 (mo) | F | Cerebrum | IHC negative for *SMARCB1* | not available | not available | + |
| ATRT6 | H | 7 (mo) | M | Cerebrum | IHC negative for *SMARCB1* | not available | not available | + |
| ATRT7 | I | 12 (mo) | F | Cerebrum | IHC negative for *SMARCB1* | not available | not available | + |
| ATRT8 | J | 17 (mo) | F | Paraspinal | IHC negative for *SMARCB1* | not available | not available | + |
| ATRT9 | L | 9 (mo) | M | Third ventricle | IHC negative for *SMARCB1* | not available | not available | +/- |
| ATRT10 | M | 3 (mo) | F | Cerebellum | IHC negative for *SMARCB1* | not available | not available | - |
| ATRT11 | N | 6 (mo) | F | Posterior fossa & pineal | IHC negative for *SMARCB1* | not available | not available | + |
| ATRT12 | O | 11 (mo) | M | Cerebrum | IHC negative for *SMARCB1* | not available | not available | +++ |
| ATRT13 | Q | 2 (mo) | F | Cerebrum | IHC negative for *SMARCB1* | not available | not available | + |
| ATRT14 | P | 7 (wk) | M | Cerebellum | IHC negative for *SMARCB1* | not available | not available | ++ |
| ATRT16 | S | 11 (mo) | F | Posterior fossa | IHC negative for *SMARCB1* | not available | not available | + |
| ATRT17 | T | 17 mo | M | Cerebellopontine angle | not available | not available | not available | + |
| ATRT18 | U | 21 (mo) | M | Cerebellum | IHC negative for *SMARCB1* | not available | not available | ++ |
| ATRT20 | V | 13 (mo) | M | Pineal region | IHC negative for *SMARCB1* | not available | not available | + |
| non_ATRT2 | W | 18 (mo) | F | Right parietal | IHC positive for *SMARCB1* | not available | not available | - |
| ATRT22 | D | 15 (mo) | F | NON-BRAIN:  Pelvic cavity | IHC negative for *SMARCB1* | Yes | not available | ++ |
| ATRT 23 | K | 18 (mo) | F | NON-BRAIN:  Renal | IHC negative for *SMARCB1* | Yes | not available | ++ |
| ATRT25 | Y | 3 (mo) | M | Cerebrellum & brainstem | IHC negative for *SMARCB1* | not available | not available | - |
| ATRT26 | Z | 12 (mo) | F | Right cranial nerves | IHC negative for *SMARCB1* | not available | not available | +++ |
| ATRT 27 | X | 5 (days) | M | NON-BRAIN: Abdomen | IHC negative for *SMARCB1* | Yes | not available | ++ |
| normal brain | normal brain | N/A | N/A | normal brain | N/A | N/A | N/A | - |
| placenta | placenta | N/A | F | placenta | N/A | N/A | N/A | + |
| cerebellum | cerebellum | N/A | N/A | cerebellum | N/A | N/A | N/A | - |

**Supplemental table 2:** Top two annotation clusters and associated terms from DAVID analysis of genes containing highly expressed LTR5_Hs or internal coding HERV-K transcripts from RNA sequencing analysis from RNA sequencing. The DAVID analysis calculates an enrichment value and associated Benjamini corrected p-value for a given gene list where a signaling pathway (term) is represented significantly more than expected by chance alone.^1,2^

| **Supplemental Table 2** | | | | |
| --- | --- | --- | --- | --- |
| **Term** | **Term Enrichment value^1^** | **Benjamini corrected p-value^2^** | **Number of genes in group** | **RNA Accession Numbers** |
| Krueppel associated box (KRAB) | 15.13 | 1.18E-  10 | 15 | 7643, 55762, 22835, 59348, 79818,  100129543, 374928, 199704, 7626,  163059, 147923, 284459, 388558, 9310, 7699 |
| zinc finger  region:C2H2type 2 | 17.72 | 6.99E-  07 | 14 | 642280, 7626, 147923, 163059, 55762,  22835, 284459, 388558, 59348,  100129543, 374928, 9310, 199704, 7699 |
| zinc finger  region:C2H2type 3 | 17.72 | 3.62E-  06 | 14 | 642280, 7626, 147923, 163059, 55762,  22835, 284459, 388558, 59348,  100129543, 374928, 9310, 199704, 7699 |
| zinc finger region:C2H2type 4 | 17.72 | 8.20E-  07 | 14 | 642280, 7626, 147923, 163059, 55762,  22835, 284459, 388558, 59348,  100129543, 374928, 9310, 199704, 7699 |
| zinc finger  region:C2H2type 5 | 16.46 | 1.30E-  06 | 13 | 7626, 147923, 163059, 55762, 284459,  388558, 59348, 100129543, 79818,  374928, 9310, 199704 |
| zinc finger  region:C2H2type 6 | 16.46 | 9.29E-  07 | 13 | 147923, 163059, 55762, 22835, 284459,  388558, 59348, 100129543, 79818,  374928, 9310, 199704, 7699 |
| zinc finger  region:C2H2type 7 | 15.19 | 1.65E-  06 | 12 | 147923, 163059, 55762, 22835, 284459,388558, 59348, 100129543, 79818,374928, 9310, 7699 |
| zinc finger  region:C2H2type 8 | 13.92 | 6.43E-  06 | 11 | 147923, 163059, 22835, 284459,  388558, 59348, 79818, 374928, 9310,  7699, 199704 |
| zinc finger region:C2H2type 9 | 13.92 | 2.75E-  06 | 11 | 147923, 163059, 22835, 284459,  388558, 100129543, 79818, 374928,  9310, 7699, 199704 |
| Neuroblastoma breakpoint  family  members  (NBPF) 1-15,20 | 5.42 | 2.32E-  05 | 4 | 149013, 284565, 400818, 200030, 200031, 200032, 200033, 200034, 200035, 200036, 200037 |
| compositionally biased region: Poly- Arg | 5.42 | 1.88E-  02 | 4 | 149013, 284565, 400818, 200037 |

**Supplemental table 3:** Terms within two annotation clusters from DAVID analysis of mass spectrometry data and their respective p values for each term and associated enrichment. The DAVID analysis calculates an enrichment value and associated Benjamini corrected p-value for a given gene list where a signaling pathway (term) is represented significantly more than expected by chance alone.^1,2^

| **Supplemental Table 3** | | | |
| --- | --- | --- | --- |
| **Term** | **Term enrichment value^1^** | **Benjamini corrected p value^2^** | **Protein Accession Numbers** |
| viral  nucleoprotein | 5.78 | 1.54E-10 | O14979, P52597, P52272, O75643,Q8WVV9, P51991, P14866, P09234,Q15029, P31942, P07910, P62314,Q09666, O43390, P08621, Q14103,P61978, Q9BUJ2, P09651, O60506 |
| viral  nucleocapsid | 5.21 | 2.30E-09 | O14979, P52597, P52272, O75643,Q8WVV9, P51991, P14866, P09234,Q15029, P31942, P07910, P62314,Q09666, O43390, P08621, Q14103,P61978, Q9BUJ2, P09651, O60506 |
| virion | 3.44 | 6.00E-04 | O14979, P52597, P52272, O75643,Q8WVV9, P51991, P14866, P09234,Q15029, P31942, P07910, P62314,Q09666, O43390, P08621, Q14103,P61978, Q9BUJ2, P09651, O60506 |
| cadherin binding involved in  cell-cell  adhesion | 4.64 | 1.34E-92 | P46940, Q7L1Q6, P62750, Q8WU90,Q9BR76, P29692, P55196, P07737,P11142, O60716, P50402, Q9UJU6,Q7Z2W4, P26196, Q9Y5X1, Q04637,Q9Y5X3, O75369, Q92522, Q15599,P62258, Q8TCG1, Q8NC51, Q99439,Q9UGI8, Q13596, P18206, Q01813,Q00587, Q12792, Q86UP2, Q5VV41,Q6PKG0, P11021, P63104, Q9Y570,Q9ULH7, P35611, P35613, P00338,P20810, O75874, P50914, P14618,O43795, P00533, P60228, Q9NR46,Q9Y6E0, P05783, Q9Y6E2, P35221,Q01970, Q9H4M9, P15311, P42166,Q06830,P42167, P49757, P15880,Q7Z478, Q9H2G2, Q15019, P21333,Q07960, Q9UHR4, Q8IUD2, Q13813, Q8WWM7, P47756, Q14C86, Q15365, P29317, Q9HC38, Q9UQB8, P25685, Q9C0C2, P40121, Q15056, P33176, Q09666, Q9P0L0, Q9NTK5, Q9NYL9, P0DMV9, P26641, Q5T0N5, P50552, P13639, O76021, Q9NQC3, Q9UPN3, Q01082, Q9UHD8, O14908, O00571, Q9H444, Q9UNF0, P61313, P61978, Q99497, Q9NUQ8, P04083, P05556, Q9Y490, P49207, O15020, Q14677, O00560, Q16643, Q86W92, O00567, P40818, Q15149, O00299, P08238, Q02878, P63244, Q9UHB6, Q9UM54, Q6P996, Q14160, P04075, P54136, Q08378, Q9H223, P30041, P52907,Q7KZF4, O95817, Q08379, Q96AG4, Q9NVD7, P50990, Q15907, O95433,Q14258, Q07157, P53990, Q92616, P49327, Q9Y371, P31939, P18031, P35241, Q32MZ4, Q16658, O60664, Q6IBS0, Q15691, P53365, P53367, Q6WCQ1, Q14247, P41091, P07355, P62820, P49959, P42224, P12931, P06733, Q9Y6W5, Q00341, Q6Y7W6, P62424, P62826, Q9Y265, Q9Y266, Q9H4G0, Q13190, P35579, O60749,  Q99961, Q05682, P42356, Q9BY44, Q14847, O60645, Q16181, Q9NZZ3, Q6P1N0, Q9H0B6, Q9UH65, P51610, Q13492, P46109, P08195, Q9UHX1, Q9H3U1, Q96C19, O96013, P46060, Q14008, Q13177, O60763, P22234, Q7KZI7, P78344 |
| cell-cell  adherens junction | 4.51 | 1.45E-88 | P46940, Q7L1Q6, P62750, Q8WU90, Q9BR76, P29692, P55196, P07737, P11142, O60716, P50402, Q9UJU6, O75955, Q7Z2W4, P26196, Q9Y5X1, Q04637, Q9Y5X3, O75369, Q92522, Q15599, P62258, Q8TCG1, Q8NC51, Q99439, Q9UGI8, Q13596, P18206, Q01813, Q00587, Q12792, Q86UP2, Q5VV41, Q6PKG0, P11021, P63104, Q9Y570, Q9ULH7, P35611, P35613, P00338, P20810, O75874, P50914,P14618, O43795, P00533, P60228, Q9NR46, Q9Y6E0, P05783, Q9Y6E2, P35221, Q01970, Q9H4M9, P15311, P42166, Q06830, P42167, P49757, P15880, Q7Z478, Q9H2G2, Q15019, P21333, Q07960, Q9UHR4, Q8IUD2, Q13813, Q8WWM7, P47756, Q14C86, Q15365, P29317, Q9HC38, Q9UQB8, P25685, Q9C0C2, P40121, Q15056, P33176, Q09666, Q9P0L0, Q15942, Q9NTK5, Q9NYL9, P0DMV9, P26641,  Q5T0N5, P50552, P13639, O76021, Q9NQC3, Q9UPN3, Q01082, Q9UHD8, O14908, O00571, Q9H444, Q9UNF0, P61313, P61978, Q99497, Q9NUQ8, P04083, P05556, Q9Y490, P49207, O15020, Q14677, O00560, Q16643, Q86W92, O00567, P40818, Q15149, O00299, P08238, Q02878, P63244,Q9UHB6, Q9UM54, Q6P996, Q14160, P04075, P54136, Q08378, Q9H223, P30041, P52907, Q7KZF4, O95817, Q08379, Q96AG4, Q9NVD7, P50990, Q15907, O95433, Q14258, Q07157, P53990, Q14254, Q92616, P49327, Q9Y371, P31939, P18031, P35241, Q32MZ4, Q16658, O60664, Q6IBS0, Q15691, P53365, P53367, Q6WCQ1, Q14247, P41091, P07355, P62820, P42224, P49959, P12931, P06733,Q9Y6W5, Q00341, Q6Y7W6, P62424, P62826, Q9Y265, Q9Y266, Q9H4G0, Q13190, P35579, O60749, Q99961, Q05682, P42356, Q9BY44, Q14847, Q16181, O60645, Q9NZZ3, Q6P1N0, Q9H0B6, Q9UH65, P51610, Q13492, P46109, P08195, Q9UHX1, Q9H3U1, Q96C19, O96013, P46060, Q14008, Q13177, O60763, P22234, Q7KZI7, P78344 |
| cell-cell  adhesion | 4.69 | 5.95E-86 | P46940, Q9UPN3, Q01082, Q7L1Q6, Q9UHD8, P62750, O14908, P29692,Q8WU90, O00571, Q9BR76, Q9H444, P55196,P07737, P11142, Q9UNF0,P61313, P61978, P50402, Q99497, Q9UJU6, Q7Z2W4, P26196, Q9NUQ8,P49207, Q9Y5X1, Q04637, Q14677, O15020, Q9Y5X3, O00560, O00567,Q16643, Q86W92, O75369, Q92522, P40818, Q96JJ3, Q15149, Q15599,P08238, P62258, Q02878, P63244, Q8TCG1, Q8NC51, Q99439, Q9UM54,Q9UHB6, Q9UGI8, Q13596, Q01813, Q00587, Q6P996, P04075, Q12792,P54136, Q86UP2, Q9H223, Q08378, P30041, P52907, Q5VV41, Q7KZF4,  Q6PKG0, O95817, Q08379, Q96AG4, P50990, O95433, P11021, Q15907, Q14258, Q07157, P53990, P63104, Q9Y570, Q9ULH7, P35611, Q92616, P35613, P00338, P49327, Q9Y371, P31939, P18031, P20810, P35241, Q32MZ4, O75874, Q16658, O60664, Q6IBS0, Q15691, P53365, P14618, P50914, P53367, O43795, P41091,  Q14247, Q6WCQ1, P60228, P07355, P62820, P42224, P49959, Q9NR46, P05783, Q9Y6E0, P06733, Q9Y6E2, Q01970, Q9Y6W5, Q9H4M9, Q00341, Q6Y7W6, P62424, P42166, P62826, Q9Y265, Q9Y266, Q06830, P42167, P49757, P15880, Q9H4G0, Q13190, Q7Z478, Q9H2G2, Q15019, Q07960, Q9UHR4, Q8IUD2, Q13813, O60749, Q8WWM7, Q99961, P42356, Q05682, P47756, Q9BY44, Q14C86, Q14847, Q15365, Q16181, O60645, Q9NZZ3, P29317, Q6P1N0, Q9HC38, Q9H0B6, Q9UQB8, P25685, Q9C0C2, Q9UH65, P51610, Q13492, Q15056, P33176, P40121, P46109, P08195, Q9UHX1, Q09666, Q9H3U1, Q9P0L0, Q96C19,O96013, P46060, Q14008, Q13177, Q9NTK5, Q9NYL9, P0DMV9, P26641,O60763, P22234, Q5T0N5, P50552, P13639, Q7KZI7, Q9NQC3, O76021, P78344 |

**Supplemental Table 4**: HML-2 transcripts encoding potential full length internal HML-2 sequences detected by primary RNA-seq analysis of AT/RT cell lines CHLA 02, CHLA 04, CHLA 05, and CHLA 06.

| **Supplemental Table 4** | | |  |  | | |
| --- | --- | --- | --- | --- | --- | --- |
| **Chromosome** | **Location*** | **Locus** | **HML-2 Strand** | **Length**  **(bp)** | **Transcript alignment to HML-2 Genes)** | **analysis** |
| 1 | 12780115-  12784947 | 1p36.21 | - | 4832 | RnaseH, RT, Protease | TEtranscripts |
| 1 | 13354553-  13361170 | 1p36.21 | + | 6617 | Protease, RT, RnaseH,  Integrase, Env | TEtranscripts |
| 1 | 150632886-  150634662 | 1q21.3 | - | 1776 | Env, Integrase, Rnase H | TEtranscripts |
| 1 | 150634650-  150634829 | 1q21.3 | + | 180 | Env | CLC-Bio/Star |
| 1 | 155627729-  155629344 | 1q22 | - | 1616 | Env | CLC-Bio/Star |
| 1 | 155629344-  155634877 | 1q22 | - | 5534 | Protease, Gag,  Integrase, RT, Rnase H | CLC-Bio/Star |
| 1 | 155629344-  155634877 | 1q22 | - | 5533 | Rec, Env, Integrase,  RnaseH, RT, Protease, Gag | TEtranscripts |
| 1 | 36490359-  36491127 | 1qp34.3 | - | 768 | Env | TEtranscripts |
| 1 | 75378054-  75380597 | 1p31.1 | + | 2543 | Gag, Protease | TEtranscripts |
| 2 | 129964635-  129964687 | 2q21.1 | - | 52 | Env | TEtranscripts |
| 2 | 186521159-  186521438 | 2q32.1 | + | 280 | Env | CLC-Bio/Star |
| 2 | 186521159-  186521438 | 2q32.1 | + | 279 | Env | TEtranscripts |
| 3 | 101692862-  101698340 | 3q12.3 | + | 5479 | Gag, Integrase, RnaseH, RT, Protease | CLC-Bio/Star |
| 3 | 101692862-  101698340 | 3q12.3 | + | 5448 | Gag, RT, RnaseH,  Integrase | TEtranscripts |
| 3 | 101698340-  101699951 | 3q12.3 | + | 1612 | Env | CLC-Bio/Star |
| 3 | 113026948-  113032475 | 3q13.2 | - | 5527 | Env, Integrase, RnaseH, RT, Protease | TEtranscripts |
| 3 | 125800278-  125800455 | 3q21.2 | - | 178 | Env | CLC-Bio/Star |

| 3 | 125800703-  125801161 | 3q21.2 | - | 459 | Integrase, Protease, Env | CLC-Bio/Star |
| --- | --- | --- | --- | --- | --- | --- |
| 3 | 125800703-  125801161 | 3q21.2 | - | 458 | Env | TEtranscripts |
| 3 | 125891263-  125898703 | 3q21.2 | + | 7440 | Gag, Protease, RT,  RnaseH, Integrase, Env | TEtranscripts |

| 3 | 185565226-  185570759 | 3q27.2 | - | 5533 | Env, Pol, Integrase,  RnaseH, Gag, Protease | TEtranscripts |
| --- | --- | --- | --- | --- | --- | --- |
| 3 | 75536830-  75537287 | 3p12.3 | + | 457 | Env | TEtranscripts |
| 3 | 75551383-  75558968 | 3p12.3 | + | 7585 | Protease, RT, RnaseH, Inegrase, Env | TEtranscripts |
| 4 | 165000233-  165001857 | 4q32.3 | + | 1624 | Env | TEtranscripts |
| 4 | 190108264-  190111540 | 4q35.2 | - | 3276 | Env, Integrase, RnaseH | TEtranscripts |
| 4 | 242385-  245212 | 4p16.3 | + | 2828 | Gag, Protease, RT, Env, Integrase | CLC-Bio/Star |
| 4 | 242385-  245212 | 4p16.3 | + | 2827 | Rt, Protease | TEtranscripts |
| 4 | 245308-  245565 | 4p16.3 | + | 258 | Integrase, RnaseH, Env | CLC-Bio/Star |
| 4 | 3978368-  3985909 | 4p16.3 | - | 7541 | Env, Integrase, RT, Protease | TEtranscripts |
| 4 | 4073397-  4073857 | 4p16.3 | + | 460 | Env | TEtranscripts |
| 4 | 9035454-  9035637 | 4p16.1 | - | 184 | Env | CLC-Bio/Star |
| 4 | 9035890-  9036346 | 4p16.1 | - | 457 | Env, Integrase | CLC-Bio/Star |
| 4 | 9035890-  9036346 | 4p16.1 | - | 456 | Env | TEtranscripts |
| 4 | 9122810-  9130255 | 4p16.1 | + | 7445 | Protease, RT, Integrase, Env | TEtranscripts |
| 4 | 9568769-  9569224 | 4p16.1 | - | 455 | Env | TEtranscripts |
| 4 | 9658974-  9666434 | 4p16.1 | + | 7461 | Env, Gag, RNAse H, Protease, RT, Integrase | CLC-Bio/Star |
| 4 | 9658974-  9666434 | 4p16.1 | + | 7460 | Protease, RT, Integrase, Env | TEtranscripts |
| 5 | 154636980-  154640034 | 5q33.2 | - | 3054 | Env, Integrase, RT | TEtranscripts |
| 5 | 154640032-  154642817 | 5q33.2 | - | 2785 | RT, Protease | TEtranscripts |
| 5 | 156660384-  156665917 | 5q33.3 | - | 5533 | Env, Integrase, RnaseH, RT, Protease | TEtranscripts |
| 5 | 93457672-  93457934 | 5q15 | - | 262 | Env | TEtranscripts |

| 5 | 93457673-  93457952 | | | | 5q15 | - | 280 | Env | |  | | CLC-Bio/Star | | |
| --- | --- | --- | --- | --- | --- | --- | --- | --- | --- | --- | --- | --- | --- | --- |
| 6 | 150859613-  150861371 | | | | 6q25.1 | + | 1759 | Env | |  | | CLC-Bio/Star | | |
| 6 | 3055163-  3055508 | | | | 6p25.2 | + | 346 | Int | |  | | CLC-Bio/Star | | |
| 6 | 3055163-  3055508 | | | | 6p25.2 | + | 345 | Integrase | |  | | TEtranscripts | | |
| 6 | 42894674-  42900373 | | | | 6p21.1 | - | 5699 | Env, Integrase, RT | | RnaseH, | | TEtranscripts | | |
| 7 | 104748902-  104752819 | | | | 7q22.2 | - | 3917 | RT, Protease, | | Gag | | TEtranscripts | | |
| 7 | 4583489-  4590929 | | | | 7p22.1a | - | 7440 | Env, Integrase, H, RT, Protease | | Rnase | |  | TEtranscripts |  |
|  |  |  |  |  |  |  |  |  |  |  |  |  |  |  |
| 7 |  | 4591993- | |  | 7p22.1b | - | 7439 |  | Env, Integrase, | Rnase |  |  | TEtranscripts |  |
|  |  | 4599432 |  |  |  |  |  |  | H, RT, Protease |  |  |  |  |  |
|  |  |  |  |  |  |  |  |  |  |  |  |  |  |  |
| 8 | 12217574-  12223144 | | | | 8p23.1 | - | 5570 | Env, Integrase, Protease | | RT, | | TEtranscripts | | |
| 8 | 12460096-  12461064 | | | | 8p23.1 | - | 968 | Env | |  | | TEtranscripts | | |
| 8 | 12461223-  12467471 | | | | 8p23.1 | - | 6248 | Env, Integrase, Protease | | RT, | | TEtranscripts | | |
| 8 | 12565087-  12565544 | | | | 8p23.1 | + | 458 | Integrase | |  | | CLC-Bio/Star | | |
| 8 | 12565087-  12565544 | | | | 8p23.1 | + | 457 | Env | |  | | TEtranscripts | | |

| 8 | 12565800-  12565977 | 8p23.1 | + | 178 | Env | CLC-Bio/Star |
| --- | --- | --- | --- | --- | --- | --- |
| 8 | 12623206-  12623664 | 8p23.1 | + | 458 | Env | TEtranscripts |
| 8 | 145022214-  145026351 | 8q24.3 | - | 4089 | Integrase, RnaseH | TEtranscripts |
| 8 | 46267760-  46271002 | 8q11.1 | - | 3242 | Integrase, RnaseH | TEtranscripts |
| 8 | 7128445-  7128897 | 8p23.1 | - | 452 | Env | TEtranscripts |
| 8 | 7498938-  7500000 | 8p23.1 | - | 1063 | Env | CLC-Bio/Star |
| 8 | 7498938-  7500000 | 8p23.1 | - | 1062 | Env, Integrase, RnaseH, Protease, Gag | TEtranscripts |
| 8 | 7500001-  7502767 | 8p23.1 | - | 2766 | Env, Integrase, RnaseH, RT, Protease, Gag | TEtranscripts |

| 8 | 7502828-  7506377 | 8p23.1 | - | 3549 | Env, Integrase, RnaseH, RT, Protease | TEtranscripts |
| --- | --- | --- | --- | --- | --- | --- |
| 8 | 8101560-  8102011 | 8p23.1 | - | 451 | Env | TEtranscripts |
| 8 | 8198204-  8205586 | 8p23.1 | + | 7382 | Env, Protease, Integrase, RT | TEtranscripts |
| 9 | 128851239-  128856449 | 9q34.11 | + | 5210 | RnaseH, Integrase, Env | TEtranscripts |
| 9 | 136781866-  136783596 | 9q34.3 | - | 1730 | Env, Integrase, RnaseH | TEtranscripts |
| 9 | 136786163-  136787981 | 9q34.3 | - | 1818 | Protease | TEtranscripts |
| 10 | 6825242-  6832673 | 10p14 | - | 7431 | Env, Integrase, RnaseH, Protease, Gag | TEtranscripts |
| 10 | 99821893-  99827959 | 10q24.2 | - | 6066 | Env, Integrase, RnaseH, RT, Protease | TEtranscripts |
| 11 | 101696031-  101699581 | 11q22.1 | + | 3550 | Gag, Protease, RT,  RnaseH, Integrase, Env | TEtranscripts |
| 11 | 110571006-  110571520 | 11q22.3 | + | 514 | Gag | TEtranscripts |
| 11 | 118721982-  118723687 | 11q23.3 | - | 1705 | Env | TEtranscripts |
| 11 | 118723687-  118729206 | 11q23.3 | - | 5519 | Env, Integrase, RnaseH, RT, Gag | TEtranscripts |
| 11 | 3448450-  3455952 | 11p15.4 | - | 7502 | Env, Integrase, Protease | TEtranscripts |
| 11 | 3544466-  3544919 | 11p15.4 | + | 453 | Env | TEtranscripts |
| 11 | 62376126-  62381099 | 11q12.3 | - | 4973 | Env, Protease,  Integrase, RT, RnaseH | TEtranscripts |
| 11 | 71675130-  71675588 | 11q13.4 | - | 458 | Env | TEtranscripts |
| 12 | 133093979-  133095471 | 12q24.33 | - | 1493 | Gag | CLC-Bio/Star |
| 14 | 24011391-  24015051 | 14q11.2 | - | 3660 | RT, Protease | TEtranscripts |
| 15 | 84160268-  84163612 | 15q25.2 | - | 3344 | RnaseH, Env | TEtranscripts |
| 16 | 2926159-  2926533 | 16p13.3 | + | 375 | Gag | CLC-Bio/Star |
| 16 | 2926505-  2926927 | 16p13.3 | + | 423 | Gag | CLC-Bio/Star |

| 16 | 2927166-  2927483 | 16p13.3 | + | 318 | Gag | CLC-Bio/Star | | |
| --- | --- | --- | --- | --- | --- | --- | --- | --- |
| 16 | 2927480-  2927660 | 16p13.3 | + | 181 | Gag | CLC-Bio/Star | | |
| 17 | 8057039-  8057470 | 17p13.1 | + | 432 | Gag | CLC-Bio/Star | | |
| 17 | 8058796-  8061901 | 17p13.1 | + | 3105 | RT | TEtranscripts | | |
| 17 | 8062292-  8063901 | 17p13.1 | + | 1610 | Integrase | CLC-Bio/Star | | |
| 19 | 27638680-  27641547 | 19q11 | - | 2868 | Env, Integrase, RNAse H |  | CLC-Bio/Star |  |
|  |  |  |  |  |  |  |  |  |
| 19 | 27638680-  27641547 | 19q11 | - | 2867 | Env, Integrase |  | TEtranscripts |  |
|  |  |  |  |  |  |  |  |  |
| 19 | 27641861-  27646453 | 19q11 | - | 4593 | Gag, Protease, RT, RNAse H | CLC-Bio/Star | | |
| 19 | 27641861-  27646453 | 19q11 | - | 4592 | RnaseH, RT, Protease, Gag | TEtranscripts | | |
| 19 | 37107697-  37114371 | 19q13.12 | + | 6674 | Env, Integrase, RnaseH, Protease | TEtranscripts | | |
| 19 | 37114452-  37115196 | 19q13.12 | - | 745 | Gag | CLC-Bio/Star | | |
| 19 | 52745023-  52748339 | 19q13.41 | - | 1209 | Env, Integrase, RnaseH | TEtranscripts | | |
| 19 | 53359095-  53360013 | 19q13.42 | + | 918 | RnaseH | TEtranscripts | | |
| 19 | 53361020-  53363705 | 19q13.42 | + | 2685 | Integrase, Env | TEtranscripts | | |
| 20 | 34129382-  34129595 | 19q13.11 | + | 214 | Gag | CLC-Bio/Star | | |
| 20 | 34130235-  34131782 | 19q13.11 | + | 1547 | RnaseH, Integrase | TEtranscripts | | |
| 20 | 34133703-  34135592 | 19q13.11 | + | 1890 | Env | CLC-Bio/Star | | |
| 22 | 18939642-  18945174 | 22q11.21 | + | 5533 | Pol, Gag, Protease, RT, Integrase, RNAse H | CLC-Bio/Star | | |
| 22 | 18939642-  18945174 | 22q11.21 | + | 5532 | Gag, Protease, RT,  RnaseH, Integrase, Env | TEtranscripts | | |
| 22 | 18945174-  18946795 | 22q11.21 | + | 1622 | Env | CLC-Bio/Star | | |
| 22 | 23537028-  23537189 | 22q11.23 | + | 162 | Gag | CLC-Bio/Star | | |
| 22 | 23538730-  23546008 | 22q11.23 | + | 7279 | Integrase, RT, RNAse H, Gag, Protease, Env | CLC-Bio/Star | | |
| 22 | 23538730-  23546008 | 22q11.23 | + | 7278 | RT, RnaseH, Integrase, Env | TEtranscripts | | |
| 22 | 23546306-  23546576 | 22q11.23 | + | 271 | Env | CLC-Bio/Star | | |
| X | 154609505-  154614774 | Xq28 | - | 5269 | Env, Integrase, RnaseH | TEtranscripts | | |
| X | 66464290-  66466342 | Xq12 | - | 2052 | Integrase, RNaseH | TEtranscripts | | |

*The base-pairs at which RNA sequence aligns with the HML-2 consensus from Dfam, The locus predicted to encode the env protein expressed in the cells is highlighted in yellow.

**Supplemental table 5:** Alignment of transcripts amplified from CHLA cell lines, cloned, and Sanger sequenced. This table includes which HERV-K gene each transcript corresponds to, the strand from which it is transcribed, and the position to which it best aligns in the human genome Hg38.

| **Sample** | **Chromosome** | **Start (F)** | **Stop (R)** | **Band** | **Transcript** |
| --- | --- | --- | --- | --- | --- |
| CHLA 02 | 1 | 75380704 | 75382515 | 1p31.1 | *np9* |
| CHLA 02 | 1 | 155627607 | 155629344 | 1q22 | *np9* |
| CHLA 04 | 1 | 155627607 | 155629344 | 1q22 | *np9* |
| CHLA 02 | 3 | 101698264 | 101700071 | 3q12.3 | *np9* |
| CHLA 02 | 3 | 125898698 | 125898838 | 3q21.2 | *rec* |
| CHLA 02 | 3 | 125898698 | 125898819 | 3q21.2 | *rec* |
| CHLA 02 | 3 | 125896744 | 125897004 | 3q21.2 | *rec* |
| CHLA 02 | 7 | 4583348 | 4585420 | 7p22.1 | *rec/ env* |
| CHLA 02 | 7 | 4591852 | 4593924 | 7p22.1 | *rec/ env* |
| CHLA 02 | 7 | 4583367 | 4585365 | 7p22.1 | *rec/ env* |
| CHLA 02 | 7 | 4591871 | 4593869 | 7p22.1 | *rec/ env* |
| CHLA 02 | 10 | 99821771 | 99821894 | 10q24.2 | *np9* |
| CHLA 02 | 11 | 118723697 | 118723738 | 11q23.3 | *np9* |
| CHLA 02 | 11 | 118721955 | 118722076 | 11q23.3 | *np9* |
| CHLA 02 | 19 | 27637448 | 27640610 | 19q11 | *rec/ env* |
| CHLA 02 | 19 | 27638558 | 27640663 | 19q11 | *rec/ env* |
| CHLA 04 | 19 | 35573749 | 35574474 | 19q13.12 | partial *env* |
| CHLA 04 | 19 | 35572413 | 35573379 | 19q13.12 | partial *env* |
| CHLA 05 | 22 | 18945874 | 18946911 | 22q11.21 | partial *env* |
| CHLA 05 | 22 | 18945147 | 18945995 | 22q11.21 | partial *env* |

**Supplemental table 6:** Statistical Analyses, replicates, and p-values for all figures.

| **Supplemental Table 4: Statistical Analyses and p-values for the figures** | | | | | |
| --- | --- | --- | --- | --- | --- |
| **Figure** | **Section** | **biological replicates** | **statistical test** | **P value** | **significance** |
| 3 | a | CHLA 02 (3 biological replicates) | Sidak’s multiple comparison test, , (t=4.120) | 0.0008 | *** |
| 3 | a | CHLA 04 (3 biological replicates) | Sidak’s multiple comparison test (t=4.175) | 0.0004 | *** |
| 3 | b | 5 biological replicates  (HERV-K LTR qPCR) | Mann-Whitney  corrected, two-tailed t-test | 0.0079 | ** |
| 3 | c | 5 biological replicates  (HERV-K LTR qPCR) | Mann-Whitney  corrected, two-tailed t-test | 0.12 | n.s. |
| 3 | d | 3 biological replicates | 2-way ANOVA | <0.0001 | **** |
| 3 | e | 3 biological replicates | 2-way ANOVA | 0.0130 | * |
| 5 | a | 3 (shRNA – compared to shRNA ENV) | Unpaired 2 tailed t-test (t=6.032) | 0.0002 | *** |
| 5 | a | 3 (CRISPRi no gRNA compared to CRISPRi +gRNA) | Unpaired 2 tailed t-test (t=4.880) | 0.0002 | *** |
| 5 | b | 2 with 5 technical replicates  (pcDNA compared to  CRISPRi 24hr | Welch's t-test | 0.0157 | * |
| 5 | b | 2 with 5 technical replicates  (pcDNA compared to  CRISPRi 48hr | Welch's t-test | 0.0029 | ** |
| 5 | b | 2 with 5 technical replicates  (pcDNA compared to  CRISPRi 72hr | Welch's t-test | 0.0159 | * |
| 5 | e | 3 biological replicates (CHLA 02 CRISPRi no gRNA compared to CRISPRi + gRNA) | Unpaired 2 tailed t-test (t=2.394) | 0.0374 | * |
| 5 | e | 3 biological replicates (CHLA 04 CRISPRi no gRNA compared to CRISPRi + gRNA) | Unpaired 2 tailed t-test (t=5.904) | 0.0021 | *** |
| 5 | f | 3 biological replicates | Unpaired t-test (t=3.232) | 0.0319 | * |
| 5 | g | 3 (CHLA 02 ENV 24hr) | One-way ANOVA, Dunnett’s multiple comparison test | 0.1185 | ns |
| 5 | g | 3 (CHLA 02 ENV 72hr) | One-way ANOVA, Dunnett’s multiple comparison test | 0.0037 | ** |
| 5 | j | 3 with at least 2 technical replicates MOI 0.1 | 2-way ANOVA | <0.0001 | **** |
| 5 | k | 3 with at least 2 technical replicates MOI 0.5 | 2-way ANOVA | <0.0001 | **** |
| 5 | l | 3 biological replicates | 2 tailed t-test (t=2.904) | 0.0440 | * |
| 5 | m | 3 biological replicates (CRISPRI no gRNA compared to gRNA) | 2 tailed t-test (t=2.895) | 0.0443 | * |
| 5 | m | 3 biological replicates (shRNA scrambled compared to shRNA ENV) | 2 tailed t-test (t=5.917) | 0.0041 | ** |
| 6 | a | 3 biological replicates (shRNA scrambled compared to shRNA (ENV) in CHLA 02) | 2-way ANOVA | 0.0004 | *** |
| 6 | b | 3 biological replicates (shRNA scrambled compared to shRNA (ENV) in CHLA 04) | 2-way ANOVA | 0.0066 | ** |
| 6 | c | 3 biological replicates | One tailed unpaired t-test | 0.0290 | * |
| 6 | d | 3 biological replicates | One tailed unpaired t-test | 0.0078 | ** |
| 6 | g | 3 biological replicates | Unpaired 2 tailed t-test | 0.0002 | *** |
| 7 | a | 3 biological replicates (3 technical replicates each) | 2-way ANOVA | 0.0011 | ** |
| 7 | b | 3 biological replicates (3 technical replicates each) | 2-way ANOVA | 0.0835 | ns |
| 7 | c | 3 biological replicates (3 technical replicates each) | 2-way ANOVA | 0.001 | ** |
| 7 | d | 3 biological replicates (3 technical replicates each) | 2-way ANOVA | 0.4124 | ns |

**Supplemental Materials and Methods**

**Immunohistochemistry on AT/RT Tissue Arrays**

Children’s Hospital of Philadelphia (CHOP) and St. Jude’s Children’s hospital provided AT/RT tissue microarrays. The mean patient age was 17.8 months, the median patient age was 11 months, and 16 females and 20 males with AT/RT were included in the tissue microarrays. The most recently collected samples were diagnosed based on the following criteria: loss of SMARCB1 expression established by immunohistochemistry, DNA sequencing, or both (Table 1). Samples obtained prior to the definition of AT/RT, including the mutation in SMARCB1, were characterized with hematoxylin and eosin (H&E) staining and immunohistochemistry for various proteins such as epithelial membrane antigen (EMA), vimentin (VIM), alpha smooth muscle actin (SMA), synaptophysin, and glial fibrillary acidic protein (GFAP) [95] (Table 1). The slides were de-paraffinized and rehydrated in the standard manner (100% Xylene for 3 min 3x, 100% ethanol for 3 min 2x, 95% ethanol for 3 min 2x, 75% ethanol for 3 min 2x, 50% ethanol for 3 min 2x, and ultra-pure water). After rehydration, antigen retrieval was performed with a sodium citrate buffer. Slides were submerged in buffer and steamed for 30 min, and then were cooled on ice for 20 min. Slides were washed with tris buffered saline with 0.05% Triton-X 100 (TBST) for 3 min 2x. Washes were followed by a treatment with a 3% hydrogen peroxide solution for blocking any nonspecific horseradish peroxidase (HRP) activity and then were treated with a blocking buffer of 1x tris buffered saline (TBS) with 0.05% triton X-100 (Millipore Sigma), and 5% donkey serum. After 1 hr of blocking, the primary antibody was diluted in blocking solution, added to the slide, and incubated, overnight at room temperature. Then, slides were washed 3x with TBST for 5 min each and the secondary antibody (Powervision (PVR) poly HRP anti-mouse IgG, PV6114) was placed on to the slides for a 2 hr room temperature incubation. Following that, the slides were washed 3 times for 5 min each and the development with 3,3′-Diaminobenzidine (DAB solution, Vector Laboratories) was performed. The solution was diluted to a 2% in 2 mL water and developed on the slides for approximately 4 min. The DAB solution was washed 3x with water for 2 min each. Next, the slides were stained with hematoxylin solution for 1.5 min and were washed again with water and then were dehydrated using the reverse order of reagents as the rehydration process. The mounting solution (Permount, Fisher Scientific) was dabbed lightly onto either side of the sample on the slide and the coverslip was mounted. The HERV-K env antibody was used at a 1:500 concentration (Austral biologicals, HERM 1855).

**Immunoblotting**

Cells were collected and stored in RIPA buffer (ThermoFisher Scientific, 89901) with 1X protein inhibitor cocktail (PIC, Promega, catalog no. P8340) to prevent protein degradation. Samples were stored at -80^ο^C until they were used for a bicinchoninic acid assay (BCA) and Western blot analysis (WB). All samples were measured with the Pierce BCA kit (ThermoFisher Scientific, 23225 and denatured with 4% SDS (sodium dodecylsulphate solution) at 95^ο^C for 10 min. After denaturation, the samples were run on a 4-12% Bis-tris gel in 3-(*N*-morpholino) propane sulfonic acid (MOPS) buffer for 1 hr at 200 mV. The protein was transferred to a polyvinylidene difluoride (PVDF) membrane (ThermoFisher, [IB24002](https://www.thermofisher.com/order/catalog/product/IB24002)) with the iBlot 2 (ThermoFisher, IB24001) machine and was blocked in 5% milk in PBST for 30 min. The membrane was then washed for 5 min 3x with PBST and incubated overnight at 4^ο^C with Vinculin (Abcam:ab129002), Cyclophilin A antibody Abcam:ab41684, HERV-K SU polyclonal (Supplemental Figure 3), or HERV-K reverse transcriptase (RT) antibody used in [96]. HERV-K SU (surface unit) Polyclonal Ab (see validation data in supplemental figure 3). Anti-Beta actin: Abcam, 8224. Anti βIII tubulin: Promega, G712A. The next morning, the primary antibody was removed, and membrane was washed for 5 min 3x with PBST. After washing, membrane is incubated with the appropriate secondary antibodies diluted in PBS with 2% non-fat milk for 1 hr. Secondary antibodies: anti-rabbit IgG, HRP linked antibody 7074 and anti-mouse IgG, HRP linked antibody 7076. Three more 5 min washes with TBST were performed and the membranes were developed by adding the horseradish peroxidase (HRP) development solution to the membrane and incubating for 5 min. Blot was then imaged with infrared on the Fluorchem Protein Simple imager and finally, its signal was quantified with ImageJ.

**RNA-Seq Analysis.**

Libraries were both prepared and sequenced at NYGC ([www.nygenome.org](http://www.nygenome.org)). For library preparation, the Illumina TruSeq Stranded Total RNA protocol was used (Illumina, San Diego, CA). Per sequencing, each library was paired-end sequenced (125 bp) for a target depth of 40 million reads (HiSeq 2500 Illumina); providing for a pair of .fastq files per library post CASAVA deplexing accessible at NCBI as GSE124210. To inspect and assure the quality of the sequence data generated, the FastQC tool was used (<https://www.bioinformatics.babraham.ac.uk/projects/fastqc/>) followed by use of the Trimmomatic tool (<http://www.usadellab.org/cms/?page=trimmomatic>) to clip away adaptor sequence, 5' bias, and/or low quality sequence that might be present. For our primary RNA sequencing analysis, surviving read pairs were mapped against the current instance of the human genome ([ftp.ensembl.org/pub/release-82/fasta/homo_sapiens/dna/](ftp://ftp.ensembl.org/pub/release-82/fasta/homo_sapiens/dna/)) two separate times using the "RNA-Seq" tool found within the CLCbio Genomics Workbench ([www.clcbio.com](http://www.clcbio.com)) under default parameters. For the first mapping, expression for ensembl annotated transcripts ([ftp.ensembl.org/pub/release-82/gtf/homo_sapiens/Homo_sapiens.GRCh38.82.chr.gtf.gz](ftp://ftp.ensembl.org/pub/release-82/gtf/homo_sapiens/Homo_sapiens.GRCh38.82.chr.gtf.gz)) was specified to be enumerated in TPM (Transcript Per Million) units. For the second mapping, expression was specified to also be enumerated in TPM units but this time for RepeatMasker "LTR" annotated features (<http://www.repeatmasker.org/>). These enumerated values were then imported into R (cran.r-project.org) and analyzed separately by mapping. To start, TPM units were pedestalled by 2, Log2 transformed, filtered to remove transcripts/features not having at least one transformed value >1, then quantile normalized. Post normalization, quality of the data was challenged and confirmed by Tukey box plot, covariance-based PCA scatter plot, and Pearson correlation heat map. To remove noise-biased expression, lowess modeling was performed across libraries (CV ~ mean expression) and the resulting fit inspected for the lowest expression value where the linear relationship between CV and mean expression was observed to be grossly lost. Expression values for a transcript/feature less than this value were subsequently construed to be noise-biased and floored to equal this value. Transcripts/Features not having at least one library with an expression greater than this value were then discarded as not detected. Expression for transcripts/features not discarded between libraries representing technical replicates were next collapsed using the max observed value per transcript/feature respectively. Where after, relationships across libraries were evaluated using expression for transcripts/features via clustered heat map (heatmap.2) and circos plot (RCircos).  <https://www.ncbi.nlm.nih.gov/geo/query/acc.cgi?acc=GSE124210>.

HERV-K internal coding sequences (HERV-K int) designate the genes encoded by the endogenous retrovirus found between the two LTRs.

**HERV-K Expression Enumeration with TEtrancripts.**

TEtrancripts utilizes unsupervised machine learning algorithms to assign reads to various loci provided in the reference file. Reference mapping of these files post adaptor clipping and quality trimming was performed by sample using STAR (<https://github.com/alexdobin/STAR>) in conjunction with a custom indexed version of the human genome (<ftp://ftp.ensembl.org/pub/release-92/fasta/homo_sapiens/dna/Homo_sapiens.GRCh38.dna.primary_assembly.fa.gz>). Specifically, known gene annotations were downloaded from Ensembl (<ftp://ftp.ensembl.org/pub/release-92/gtf/homo_sapiens/Homo_sapiens.GRCh38.92.gtf.gz>) then augmented to include two additional sets of HERV-K related annotations: 1) UCSC RepeatMasker regions annotated with “repClass” matching “LTR” and “repFamily” matching “ERVK” (<http://genome.ucsc.edu/cgi-bin/hgTables>), 2) HERV-K coding regions identified by BLAST (<https://blast.ncbi.nlm.nih.gov/Blast.cgi>) to have homology (E < 1e-6) with consensus sequences for HERV-K env, gag, pol, pro, RNAse H, or rt-Pol (<http://www.dfam.org/home>). This augmented annotation file was then passed to the “genomeGenerate” command supported in STAR to generate a custom indexed version of the human genome. Post mapping to this custom indexed genome, one alignment file was produced per sample in .bam format. Alignment files were next sorted by read name using the “sort” command supported in samtools (<https://github.com/samtools/samtools/releases>) then passed by sample to the “TEcount” function supported in the TEtoolkit (<https://github.com/mhammell-laboratory/tetoolkit/issues>) to produce expression counts for each HERV-K related annotation. These expressions were finally imported into R (<https://cran.r-project.org/>) and summarized by circos plot using the RCircos() function.  Sequence data is available for download from NCBI GEO: <https://www.ncbi.nlm.nih.gov/geo/query/acc.cgi?acc=GSE124210>

Both sets of technical replicates are included in the Circos diagrams for the TEtranscripts analysis to display the similarity between libraries.

**Chromatin Immunoprecipitation and qRT-PCR**

Prior to collection for immunoprecipitation AT/RT cells were grown in a T25 flask at a density of 5 million cells per 10 mL media for 48 hr before collection. After 48 hr of growth, cells were collected and resuspended in fresh media. Paraformaldehyde was added to a final concentration of 1% and sample incubated at room temperature for 10 min while mixing. Crosslinking reaction was stopped by the addition of 1.25 M glycine to a final concentration of 0.125 M. Cells were rocked at room temperature for 30 min. Cell pellets were collected by centrifugation at 2,000 RPM for 5 min. Pellets were then frozen on dry ice and stored in the -80^ο^C freezer until ChIP was performed. To begin ChIP, cell pellets were thawed on ice, 1 mL of ChIP lysis buffer was added, cells were resuspended and incubated on ice for 10 min. Cells were then spun at 5,000 RPM at 4^ο^C for 5 min for nuclei isolation. Supernatant was discarded and pellet was resuspended in 300 μl of nuclei lysis buffer. Samples incubated on ice for 10 min. Chromatin was then sonicated to an average length of 500 bp with 3 pulses at 25% power for 10 sec with 30 sec rests between cycles. Sheared chromatin was then centrifuged at 14,000 RPM for 10 min at 4^ο^C to remove debris. Supernatant was removed from Eppendorf tube and diluted 5-fold with ChIP dilution buffer (e.g. 300 μl of sample + 1200 μl ChIP dilution buffer). 100 μl of diluted solution was removed and frozen at -80^ο^C to use for “input” sample later.

For the ChIP experiment 350 μl of previously diluted sample was incubated with 30 μl of dynabeads (ThermoFisher Scientific, Catalog 10004D), 2.5 μg of antibody, and 120 μl additional dilution buffer at 4^ο^C on a rotating rack for 14 hrs. The following day, the Eppendorf tubes were placed on a magnetic rack and the supernatant was removed. The beads were then washed with 1000 μl of low salt buffer three times. Two washes with high salt buffer and two washes with TE buffer were then performed. Finally, the TE was removed from the beads and 200 μl of elution buffer was added. Tubes were placed on the thermomixer at 65^ο^C with a speed of > 1,000 RPM for 30 min. Tubes were removed from thermomixer, supernatant was put into a new Eppendorf tube, and 4 μl of proteinase K (20 mg/mL) was added to the solution. Samples were left on thermomixer for 2 hrs shaking at 55^ο^C > 1,000 RPM. The input removed at the beginning of the protocol was also thawed, elution buffer was added, and samples were placed on thermomixer for 30 min at 65^ο^C. Input samples also received 4 μl of proteinase K (20 mg/mL) and were left to shake alongside the other samples at 55^ο^C for 2 hrs. Finally, a phenol chloroform extraction was performed to isolate the immunoprecipitated DNA. 400 μl of phenol isoamyl alcohol (ThermoFisher Scientific, 15593031) was added to the eluate and sample was vortexed for 15 sec and spun at 4^ο^C for 10 min at 14,000 RPM. 350 μl supernatant was removed and 400 μl of chloroform was added to each sample. Samples were vortexed for 15 sec and spun at 14,00 RPM for 10 min at 4^ο^C. 300 μl of supernatant was removed from each sample and added to 300 μl ice-cold isopropanol plus 3 μl of glycerol. Samples were incubated at -80^ο^C in freezer for 1 hr and then spun at 14,000 RPM for 10 min. Supernatant was removed without disturbing the DNA pellet, and the pellet was washed with 500 μl ice-cold 80% ethanol and then spun at maximum speed for 5 min. Supernatant was removed and pellet was dried for 5 min before being resuspended in 35 μl of DNase/RNase free H_2_O. To obtain the ratio of specific sequences pulled down during ChIP, semi-quantitative PCR was performed using primers that spanned the HERV-K transcription start site (TSS): LTR Forward (5'-GTT TGT CTG CTG ACC CTC TC-3') and Reverse (5’-AGC CTC TGA GTT CCC TTA GT-3’); qPCR was also performed using primers for an unrelated genomic region (hypoxanthine phosphoribosyltransferase 1; HPRT1), Forward (5’-GCT GAC CTG CTG GAT TAC AT-3’) and Reverse (5’-GGT TTG CAG AGA TTC AAA GAA-3’). Results are shown as percent of input chromatin, calculated using the formula % input = 100*2^(Ct^_[input]_ – ^Ct^_[IP]_). Antibodies used for ChIP: Go-ChIP grade purified Anti-RNA polymerase II antibody, 904004, Biolegend. Mouse Mab IgG XP (R) isotype control antibody, Cell Signaling, 3900S. Ini1 antibody (A-5): sc-166165 (Ini1 is another name for SMARCB1), Santa Cruz.

For the Myc ChIP in the 293T cells and in the CHLA 02 cells the aforementioned procedures were also followed using the C-Myc antibody from abcam catalog # ab17355.

**Biotin-Streptavidin DNA-protein immunoprecipitation assay**

Purified and active recombinant C-Myc protein (500 ng) (abcam ab169901) was incubated for 15 min at room temperature with 30 uL of Dynabeads^TM^ M-270 (Invitrogen) and 1 ug of biotinylated nucleotides corresponding to different sequences in the HERV-K LTR in HML-2 loci present at 7p22.1a and 7p22.1b (see chart below).

| **oligo name** | **sequence** |
| --- | --- |
| Nt_139_144 | CCCCCAAC**CCCGTG**CTCTCT |
| Nt_154_160 | CTCTGAA**ACATGTG**CTGTGT |
| Nt_350_355 | TTCTCC**CCATGTG**ATAGCCT |
| Nt_374_379 | ATATGGC**CTCGTG**GGAAGGG |
| Nt_709_714 | CCTTTGTT**CACATG**TTTGTC |
| Nt_939_944 | AACACCC**ACAGGTG**TGTAGG |
| non-targeting | GTATTACTGATATTGGTGGG |
| scrambled_myc_site | ATCCCCCTGCACCTCCTCCG |
| scrambled_2_myc_site | TACAATCGTTCGTTCTCCCG |

The beads were then washed three times with a low stringency buffer (PBS,137 mM NaCl), twice with a high stringency buffer (10mM Trid, PH 7.5, 1 mM EDTA, 300 mM NaCl), and once with 1X PBS. The bound protein was eluted from the beads with 1X SDS loading buffer at 95C for 10 minutes and the elutions were loaded in equal volumes on a 4-12% (w/v) Bis-Tris electrophoresis gel (Life technologies). Proteins were then transferred to a PVDF membrane and immunoblotted with an antibody against C-Myc (ab17355).

**Verification of the specificity of the HERV-K envelope antibody**

To verify the specificity of the HERV-K env antibody, a depletion experiment was performed. The antibody was incubated overnight with recombinant HERV-K Env protein and a western blot was made with three different protein lysates of Hela cells. The blots were stained with the depleted and the undepleted antibody and the pattern of bands was compared. In the blot stained with the regular antibody, a doublet band appeared at the correct size of the HERV-K Env. No band was visible in the blot stained with the depleted antibody (Supplemental figure 2A). To verify that the antibody could efficiently detect variations in the levels of HERV-K Env produced from different genomic loci, Hela cells were transfected with an empty vector (pcDNA), with DNA constructs encoding 3 different genomic loci of HERV-K env (located in chromosome 11, 12 and 19), or with a construct encoding a consensus sequence of HERV-K env. A clear increase in both the full-length protein and in the transmembrane subunit were detected in the cells transfected with each of the HERV-K env sequences compared to pcDNA-transfected cells (Supplemental figure 2B). To clarify the nature of the doublet band at 85 kDal, protein lysates of human neural stem cells and mouse neural stem cells were run on an agarose gel, transferred to a membrane and stained with the HERV-K Env antibody. Although HERV-K is not present in the mouse genome, the upper band of the doublet does appear in the mouse cells lysate, while the lower band is not present (Supplemental figure 2C). Thus, we concluded that the lower band is the only one which is HERV-K Env-specific. To further confirm the specificity of the lower Env band, CHLA 02 cells were transfected with a lentiviral construct with gRNA targeting HERV-K LTR5_Hs and one construct without gRNA. The expression of the lower band of HERV-K Env decreases with the gRNA in two biological duplicates as compared to Beta Actin (Supplemental figure 2D). ShRNA targeting HERV-K Env transcripts also led to a significant decrease in the lower band in two biological duplicates compared to Beta Actin expression (Supplemental figure 2D).

**Full Western blot images**

| **Primer name** | **primer sequence** |
| --- | --- |
| Chr7_p22_LTR_Myc_F1 | TTCTTCTGCCTTGAGATTCTGTT |
| Chr7_p22_LTR_Myc_R1 | CACCGCCCTTAATCCATTT |
| Chr7_p22_LTR_Myc_F2 | TCTATGACCTTACCCCCAACC |
| Chr7_p22_LTR_Myc_R2 | TCTGTTTAACAAAGCACATCCTG |
| Chr7_p22_LTR_Myc_F3 | AAGCCAGGTATTGTCCAACG |
| Chr7_p22_LTR_Myc_R3 | CTCCTCAGCACAGACCCTTT |
| Chr7_p22_LTR_Myc_F4 | GGAAAGCCAGGTATTGTCCA |
| Chr7_p22_LTR_Myc_R4 | CTCCTCAGCACAGACCCTTT |
| Chr7_p22_LTR_AR_F1 | TGCATATCTAAAAGCACAGCA |
| Chr7_p22_LTR_AR_R1 | GTGGGTGTTTCTCGAAGAGG |
| Chr7_p22_LTR_AR_F2 | TGCATATCTAAAAGCACAGCACT |
| Chr7_p22_LTR_AR_R2 | TCATCTGTGGGTGTTTCTCG |
| Chr7_p22_LTR_Myc_F5 | TCTCTGTGTCTTTTTCTTTTCCAA |
| Chr7_p22_LTR_Myc_R5 | AGGGGTGGGTTGCCCCTA |
| HPRT_gDNA_ChIP_F | GCTGACCTGCTGGATTACCAT |
| HPRT_gDNA_ChIP_R | GGTTGGCAGAGATTCAAAGAA |
| LTR_TSS_F | GTTTGTCTGCTGACCCTCTC |
| LTR_TSS_R | AGCCTCTGAGTTCCCTTAGT |
| HK_envelope_F2 | CTGCCAAACCTGAGGAAGAA |
| HK_envelope_R2 | ACCAACCAATTTTGGACTGC |
| HK_polymerase_F | TCACATGGAAACAGGCAAAA |
| HK_polymerase_R | AGGTACATGCGTGACATCCA |
| HK_gag_R | AGCAGGTCAGGTGCCTGTAACATT |
| HK_gag_R | TGGTGCCGTAGGATTAAGTCTCCT |
| ACTB_F | ATCGAGCACGGCATCGTCA |
| ACTB_R | AGCACAGCCTGGATAGCAAC |
| N-Ras_F | CAGAGGCAGTGGAGCTTGA |
| N-Ras_R | GCTTTTCCCAACACCACCT |
| HPRT_F | CATTATGCTGAGGATTTGGAAAGG |
| HPRT_R | CTTGAGCACACAGAGGGCTACA |

| **List of Antibodies/ recombinant proteins** | | | |
| --- | --- | --- | --- |
| Name | Manufacturer | Concentration Used | Catalog number |
| C-Myc protein | Abcam | 2 uL (diluted 1 in 10) per pulldown | ab17355 |
| Anti-RNA polymerase II antibody | Biolegend | 5 ug | 904004 |
| Mouse Mab IgG XP (R) isotype control antibody | Cell Signaling | 5 ug | 3900S |
| Ini1 antibody (A-5): | Santa Cruz | 5 ug | sc-166165 |
| C-Myc antibody | Abcam | 5 ug | ab17355 |
| Vinculin antibody | Abcam | 1:1000 | ab129002 |
| Cyclophilin A antibody | Abcam | 1:1000 | ab41684 |
| HERV-K SU antibody |  | 1:1000 | QRKAPPRRRRHRNRC (HERV-K env amino acid position:8-21), CSDLTESLDKHKHKK (env amino acid position:294-307), and CSKRKGGNVGKSKRD (env amino acid position: 680-693). |
| B-actin antibody | Abcam | 1:1000 | 8224 |
| βIII tubulin antibody | Promega | 1:1000 | G712A |
| HERV-K Env antibody | Austral Biological | 1:500 | HERM 1855 |
| HERV-K TM Env antibody (PAb) |  | 1:1000 | Peptide used to generate antibody: CSKRKGGNVGKSKRD |
| anti-rabbit IgG, HRP linked antibody | Cell Signaling | 1:2500 | 7074 |
| Anti-mouse IgG, HRP linked antibody | Cell Signaling | 1:2500 | 7076 |
| Poly-HRP anti-mouse IgG | Powervision | 1:2500 | PV6114 |
| Anti-CD98 | Abcam | 1:200 | ab108300 |
| Purified Tubulin B 3 (TUBB3) antibody | Biolegend | 1:1000 | 801213 |
| OCT4 antibody | Millipore sigma | 1:200 | AB3209 |
| Nestin antibody | Millipore sigma | 1:200 | AB5326 |
| Pax6 (1C8) antibody | Novus biologicals | 1:200 | NBP1 51622 |
| Goat Rabbit IgG antibody | Alexa fluor | 1:500 | ab150116 |
| Goat mouse IgG antibody | Alexa fluor | 1:500 | ab150080 |
